# Random matrix theory tools for the predictive analysis of functional magnetic resonance imaging examinations

**DOI:** 10.1117/1.JMI.10.3.036003

**Published:** 2023-06-14

**Authors:** Derek Berger, Gurpreet S. Matharoo, Jacob Levman

**Affiliations:** a St. Francis Xavier University, Department of Computer Science, Antigonish, Nova Scotia, Canada; b St. Francis Xavier University, ACENET, Antigonish, Nova Scotia, Canada; c St. Francis Xavier University, Department of Physics, Antigonish, Nova Scotia, Canada; d Athinoula A. Martinos Center for Biomedical Imaging, Charlestown, Massachusetts, United States; e Nova Scotia Health Authority, Research Affiliate, Antigonish, Nova Scotia, Canada

**Keywords:** random matrix, spectral rigidity, level number variance, functional magnetic resonance imaging, classification, machine-learning

## Abstract

**Purpose:**

Random matrix theory (RMT) is an increasingly useful tool for understanding large, complex systems. Prior studies have examined functional magnetic resonance imaging (fMRI) scans using tools from RMT, with some success. However, RMT computations are highly sensitive to a number of analytic choices, and the robustness of findings involving RMT remains in question. We systematically investigate the usefulness of RMT on a wide variety of fMRI datasets using a rigorous predictive framework.

**Approach:**

We develop open-source software to efficiently compute RMT features from fMRI images and examine the cross-validated predictive potential of eigenvalue and RMT-based features (“eigenfeatures”) with classic machine-learning classifiers. We systematically vary pre-processing extent, normalization procedures, RMT unfolding procedures, and feature selection and compare the impact of these analytic choices on the distributions of cross-validated prediction performance for each combination of dataset binary classification task, classifier, and feature. To deal with class imbalance, we use the area under the receiver operating characteristic curve (AUROC) as the main performance metric.

**Results:**

Across all classification tasks and analytic choices, we find RMT- and eigenvalue-based “eigenfeatures” to have predictive utility more often than not (82.4% of median AUROCs>0.5; median AUROC range across classification tasks 0.47 to 0.64). Simple baseline reductions on source timeseries, by contrast, were less useful (58.8% of median AUROCs>0.5, median AUROC range across classification tasks 0.42 to 0.62). Additionally, eigenfeature AUROC distributions were overall more right-tailed than baseline features, suggesting greater predictive potential. However, performance distributions were wide and often significantly affected by analytic choices.

**Conclusions:**

Eigenfeatures clearly have potential for understanding fMRI functional connectivity in a wide variety of scenarios. The utility of these features is strongly dependent on analytic decisions, suggesting caution when interpreting past and future studies applying RMT to fMRI. However, our study demonstrates that the inclusion of RMT statistics in fMRI investigations could improve prediction performances across a wide variety of phenomena.

## 1 Introduction

Though first developed to describe the fluctuation of nuclei energy levels in quantum physics,^1^^,^^2^ Random matrix theory (RMT) has been shown to have extremely broad potential. In small-scale physical systems, RMT universalities have been observed in quantum chaotic systems, complex nuclei, atoms, molecules and disordered mesoscopic systems;^1^^–^^7^ at larger scales, RMT has been applied to atmospheric physics,^8^ stock cross-correlations,^9^ social networks,^10^ random networks,^11^ network-formation in liquids,^12^^,^^13^ and amorphous clusters.^14^^–^^16^ Within biological systems, RMT has also been used to successfully model aspects of amino acid functional relationships,^17^ synchrony in epileptic seizures,^18^ and in protein–protein interactions both in different species^19^ and breast cancer.^20^ RMT has also been used to guide statistical decisions in principal components analyses^21^^–^^23^ and, more recently, has provided insights into the behaviors of deep neural networks.^24^^,^^25^

At its most rudimentary, RMT describes the expected behavior of the eigenvalues—also often called the spectra or levels—of a number of classes of random matrices.^1^^,^^26^^,^^27^ A random matrix is a matrix where each entry is a random variable. Typically, each random variable is independent and identically distributed (iid), however, modern extensions allow for some dependence.^28^ A class or ensemble of random matrices can be defined by the type of iid distribution involved. For example, the Gaussian orthogonal ensemble (GOE^1^^,^^2^) is a “class” or “ensemble” of random matrices that comprises orthogonal matrices with each entry being sampled from a standard Gaussian, and the Marchenko–Pastur distribution describes the limiting behavior of the singular values of iid rectangular random matrices where the iid distribution is arbitrary.^1^ RMT often finds that in the infinite limit, the expected eigenvalue distribution of a number of classes of random matrices can be described quite precisely.^1^^,^^26^^,^^27^

Of course, given a particular non-random matrix or instantiation of a random matrix, one can, in general, only estimate the likelihood that it is a member of a certain class or ensemble since certain RMT ensembles (such as the GOE) can yield arbitrary matrices with extremely low probability. Thus RMT also includes statistical measures (“spectral observables”) and tools to help compare empirical observations to theory.^1^^,^^2^

When or if RMT has real-world explanatory potential, this will most likely be when dealing with a complex system of many (hundreds or more^1^) interacting components, rather than a system that is too small for statistical regularities to reliably surface. If such a system has a matrix representation, and the eigenvalues and spectral observables of this representation have distributions similar to those predicted by RMT, it suggests that the system is either highly random or chaotic. By contrast, if the observed spectra deviate significantly from RMT-predicted spectra, this suggests otherwise. A number of studies have used RMT to make such interpretations and comparisons between systems.^8^^,^^10^^,^^11^^,^^19^^,^^20^^,^^29^^–^^32^

### 1.1 RMT and Neurobiological Signals

In the human brain, each neuron, collection of neurons, or region of interest (ROI) is a potentially interacting component in a complex system. RMT may have potential in describing the totality of these interactions, provided that measurements of functioning can be obtained with sufficient spatial and temporal resolution to speak to some neurobiological or neuropsychological phenomenon of interest.

For an imaging modality like functional magnetic resonance imaging (fMRI), where changes in the blood-oxygenation-level-dependent (BOLD) signals are related to neural activity, RMT may be an ideal starting point, as each voxel time course (or collection of such time sources, i.e., ROIs) can be considered an interacting component of the system. Likewise, in functional connectivity analyses, statistical relationships of the BOLD ROI time courses are investigated in the hope of gaining insights into brain function (see Refs. 33 and 34 for reviews, but also Refs. 35 and 36 for challenges facing functional connectivity analyses). In this framework, each connection or correlation can be considered a system component, and the eigenvalues of such a correlation matrix can be examined from the perspective of RMT.

The earliest study taking this approach demonstrated that spectra of the correlations between electroencephalographic signals closely resemble those of the GOE.^29^ In fMRI, RMT has been used to evaluate the quality of whole brain features extracted from fMRI data,^37^^,^^38^ and in diffusion MRI to aid in the selection of the number of components to employ in principal-component reduction analysis and denoising.^22^^,^^23^^,^^38^

RMT has also been used in ROI-based fMRI functional connectivity studies to investigate differences between rest and task states,^30^ between subjects with and without attention-deficit hyperactive disorder (ADHD),^31^ between pain and non-pain states,^32^ and between left-sided versus right-sided motor imagery.^39^ Although no differences were found in the latter study, across the first three studies, the spectra of resting or low-attention states exhibited properties close to the GOE. These findings suggest that certain aspects of psychological processes might be characterized, in part, by features computed from the eigenvalues of fMRI correlation matrices, and that these features might vary in an interpretable manner across psychological processes. If this is the case, RMT could aid in understanding the functioning of the human brain.

### 1.2 Eigenvalue Features

The basic insight of RMT is thus that eigenvalues alone may provide interesting information about highly complex systems. However, real systems are usually noisy and involve a mixture of random and non-random components and interactions. RMT is statistical in nature, describing only the expected behavior of the spectra of iid random matrices. To this end, a number of summary statistics or “spectral observables”^1^^,^^2^ can be computed from empirically observed spectra, with these spectral observables sometimes being better suited for further analysis, or for the comparison of empirical observations to theory. Two such summary statistics that have been popular^8^^,^^10^^,^^11^^,^^19^^,^^20^^,^^29^^–^^32^ are the “spectral rigidity” and “level number variance” (“rigidity” and “level variance” for short; details in Sec. 3).

However, from a predictive standpoint, these summary statistics may mask predictively useful information. If the basic RMT insight is that the eigenvalues alone can provide understanding of a system, then those eigenvalues (or other simple transformations of them) ought also to be predictively useful. We examine a number of such features in this study and refer to both RMT-derived features, such as the rigidity and level variance, and non-RMT-derived features collectively as “eigenfeatures” in this study.

### 1.3 Functional Connectivity Reduction

The functional connectivity—often, the matrix of correlations of various collections of voxels of an fMRI scan—is *a priori* a useful representation of the fMRI data. However, the full correlation matrix between all voxels is itself often computationally infeasible to work with, and thus is reduced in various ways prior to being used in analyses.

Typical reductions include the use of a “seed” voxel or collection of voxels:^40^^,^^41^ the mean signal over that ROI is correlated with all other N ROIs of interest to generate a reduced functional connectivity matrix (or image) with one correlation value at each non-seed ROI. This sort of reduction reduces the functional connectivity to N values but necessarily biases the analysis to the seed ROI.

Likewise, one can work with ROI mean signal reductions, or independent component analysis reductions,^40^^,^^41^ and work with the N×N matrix of the correlations of these reductions. This analysis is, *a priori*, sensitive to the choice of ROIs, and, in the case of anatomical atlases/parcellations for ROIs, may also lack theoretical justification.

RMT suggests a potentially useful reduction in the form of the eigenvalues of the voxelwise functional connectivity matrix. An N×t matrix of N time series of length t, and where t≪N will have a symmetric correlation (or covariance) matrix with t−1 non-zero, positive real-valued eigenvalues. These eigenvalues can be computed highly efficiently via transposition of the voxelwise correlation or covariance matrix.

### 1.4 Limitations of Previous Work

Previous studies applying RMT to functional connectivity data^31^^,^^32^^,^^39^ took largely descriptive/explanatory approaches, noting only differences in RMT across subgroups and/or conditions. For example, Wang et al.^31^ and Gu et al.^39^ used a Kolmogorov–Smirnov test, and Wang et al. used a t-test to provide evidence of differences in various RMT metrics. However, statistically significant differences in a metric do not necessarily imply practical significance or predictive utility,^42^ nor do they imply generalization or replicability.^43^ Cross validation, by contrast, attempts to more directly assess these properties.^44^

In addition, the previous papers do not provide publicly accessible code to reproduce results. The extraction of the spectral rigidity and level variance is computationally demanding and mathematically and algorithmically non-trivial and additionally requires an “unfolding” procedure.^1^^,^^2^ Unfolding is an exponential fitting procedure that involves a number of highly subjective decisions regarding outliers and the flexibility of the fitting function. These decisions are, unfortunately, often poorly documented or even entirely missing from method descriptions, despite being known to often dramatically impact RMT conclusions.^45^^–^^49^

In addition to the flexibility in the implementation and application of RMT to empirical data, there is also flexibility introduced by analytic choices made in the complex preprocessing pipelines of fMRI.^50^ These “researcher degrees of freedom,”^51^ in combination with the absence of reproducible code and data availability, and predominance of descriptive and explanatory approaches, may raise doubts about the basic robustness and practical utility of past RMT-based functional connectivity analyses.

What is missing is a rigorous, systematic, reproducible investigation of the predictive value of RMT metrics across a wide variety of data, analytic choices, RMT features, and preprocessing decisions, with RMT features being compared to simpler alternative baseline predictors. The current study aims to remedy this.

## 2 Datasets

### 2.1 Overview

We selected fMRI data publicly available on the OpenNeuro platform.^52^ Selection criteria were somewhat subjective, but we required each dataset to (1) comprise 10 or more human subjects, (2) have all fMRI runs have the same spatial and temporal resolutions, (3) have fMRI images that can be split into various classes for a number of binary classification tasks, and (4) allow reasonable classification of each run using all of the run 4D voxel data only. That is, for this last point, classification of a run should be reasonable in the absence of run-specific task or event timings or details and should not require using only some subset of the total set of 3D volumes (e.g., those associated only with some task or event timings). This yielded seven datasets total.

In order not to inundate readers with dataset details and because this is an exploratory investigation of RMT, which is not committed to any specific theory (In fact, datasets and, later, the classification tasks were chosen without investigation into any findings or publications associated with the data, both in order to reduce bias in our decisions, and because the original authors analytic/statistical approaches and intentions have very limited relevance to our whole-brain, voxelwise, multiverse predictive approach.), we refer the reader to the original publications and/or data releases when greater detail is needed and highlight only the most basic aspects of each dataset here. Dataset scan parameters and acquisition details are summarized in Table 1, and sample and subgroup sizes are summarized in Table 2.

**Table 1:** Included fMRI dataset details. Name, identifier for paper. Dimensions listed as M×N×P, indicate P axial slices each with dimensions M×N. TR, time of repetition (s). Volumes, number of 3D volumes per run. n_scans, total number of 4D images in dataset (number of subjects times number of runs per subject).

Name	Dimensions	Voxel size (mm)	TR	Volumes	n_scans
Aging	74 × 74 × 32	3.0 × 3.0 × 4.0	2.0	300	62
Bilingual	100 × 100 × 72	1.8 × 1.8 × 1.8	0.88	823	90
Depress	112 × 112 × 25	2.0 × 2.0 × 5.0	2.5	100	72
Learn	64 × 64 × 36	3.0 × 3.0 × 3.0	2.0	195	432
Osteo	64 × 64 × 36	3.4 × 3.4 × 3.0	2.5	300	74
Park	80 × 80 × 43	3.0 × 3.0 × 3.0	2.4	149	552
Attention	128 × 128 × 70	1.5 × 1.5 × 1.5	3.0	300	90

**Table 2:** Sizes and other details for classification task subgroups. ID, Identifier for paper; subgroup, name of subgroup used in classification task; subjects, number of subjects; task, fMRI task; ANT, ANT.^53^

ID	Subgroup	Subjects	Task	Scans per subject
Aging	Older	28	Resting-state	1
	Younger	34	Resting-state	1
Bilingual	Bilingual	59	Resting-state	1
	Monolingual	33	Resting-state	1
Depress	Depression	51	Resting-state	1
	Control	21	Resting-state	1
Learn	Task	24	Learn image sketches	16
	Rest	24	Resting-state	2
Osteo	Duloxetine	19	Resting-state	1
	Pain	37	Resting-state	1
	Nopain	20	Resting-state	1
Park	Parkinson’s	25	ANT	12
	Control	21	ANT	12
Attention	Vigilant	11	Resting-state	2
	Non-vigilant	11	Resting-state	2
	Trait-attentive	11	Resting-state	2
	Trait-non-attentive	11	Resting-state	2
	Task-attentive	11	Resting-state	2
	Task-non-attentive	11	Resting-state	2

### 2.2 Datasets and Classification Tasks

The “aging” dataset^54^ included rs-fMRI data for 34 subjects with a mean age of 22 years (range: 18 to 32 years) and 28 subjects with a mean age of 70 years (range: 61 to 80 years). For our analysis, we make the binary classification task the prediction of subject age-group membership, i.e., younger v older.

The “bilinguality” dataset^55^^,^^56^ examined English and Spanish-speaking monolinguals and multilinguals during a prolonged resting state. The original study grouped participants into three subgroups: early versus late bilinguals versus monolingual controls.^56^ For our analysis, we make the binary classification task monolingual v bilingual.

The “depression” dataset^57^^,^^58^ includes rs-fMRI scans from non-depressed controls and mildly or moderately depressed subjects. In our analysis, the binary classification task is depress v control.

The “learning” dataset^59^^,^^60^ has both rs-fMRI and task fMRI scans available for all subjects, and task v rest is chosen as the binary classification task for our analysis. The task is complex and difficult to summarize here adequately, but it involved multiple phases where subjects were presented with various related abstract images, and then later tested for their memory of certain aspects of the learned images. Thus the classification task might also be considered learning v rest.

The “osteo” dataset^61^ includes whole-brain rs-fMRI scans of healthy, pain-free controls (nopain condition), and individuals with knee osteoarthritis. Osteoarthritic patients were treated for two weeks with either placebo (pain condition) or duloxetine (duloxetine condition). We use the three binary classification tasks nopain v duloxetine, nopain v pain, and pain v duloxetine for our analysis.

The “park” dataset^62^ includes subjects with non-demented Parkinson’s disease and healthy controls performed a number of repetitions of the attention network task (ANT^53^) during scans. The classification task for this dataset is Parkinson’s versus controls, i.e., park v ctrl.

The “attention” dataset^63^ is a high-resolution rs-fMRI dataset including a battery of psychological measures for each subject. Since previous studies employing RMT sometimes interpreted their findings with respect to attentional processes,^31^^,^^32^ we divided subjects into various high versus low attention binary classification tasks based on median splits of subsets of the metrics available in this study.

The vigilant v nonvigilant task was formed based on self-report questionnaire items involving “vigilance.”^64^ The trait_attend v trait_nonattend task was formed using PANAS-X^65^ items related to self-reported wakefulness and attention over the past weeks. Finally, the task_attend v task_nonattend classification task was constructed using the scores of the Conjunctive Continuous Performance Task.^66^ This is a behavioral performance task that requires the subject to quickly and selectively respond to only certain visual stimuli. Details allowing exact reproduction of these splits are available in this paper’s source code.

## 3 Methods

### 3.1 fMRI Preprocessing

We limited preprocessing to a simple pipeline of, in order: (1) brain extraction, (2) slice timing correction, (3) motion correction, and (4) registration to the MNI152.^67^^,^^68^ 2mm isotropic, asymmetric template version C made available through TemplateFlow.^69^

Brain extraction was performed first with BET^70^ and then with ANTs^71^ to clean up residue left behind by BET. BET results were visually inspected for each image in each dataset, and BET fractional intensity threshold (-f argument) values were modified to ensure brain extraction was acceptable. Motion-correction was performed with FSL’s MCFLIRT^72^ and slice time correction with FSL’s slicetimer^73^ tool. Finally, functional images were registered directly to the MNI152 template via ANTs.^71^

We limited preprocessing methods to these steps because data required for other typical preprocessing steps (e.g., field intensity maps and physiological measurements) was missing, but also because all pipeline intermediates were saved to later compare the effect of increasing degrees of preprocessing on feature predictive utility (see Sec. 3.6), and it was important to limit the number of preprocessing steps to prevent excessive computational costs and comparisons.

### 3.2 Feature Extraction

When dealing with large inputs, any predictive analysis must reduce that input into predictively useful features. Any method that reduces a larger set of data is potentially valid as a feature extraction method, and RMT, which summarizes matrix data by summarizing the eigenvalue distributions, may be useful for feature extraction. However, different feature extraction methods may have different advantages and disadvantages in terms of the computational complexity, amount of summary or selection involved, and in interpretability. RMT is both mathematically and computationally complex, and thus RMT-based features should be compared to alternate features that may be easier to understand or compute.

Thus for each preprocessing degree and fMRI image, features were extracted from all N non-constant brain voxels, where N may differ for each image. After this process, each scan yields an N×t matrix M, where t is the number of volumes acquired. All features extracted summarize this matrix M.

#### 3.2.1 Raw eigenvalues

The computing time and memory requirements for calculating the full N×N matrix M of Pearson correlation coefficients (and subsequent eigenvalues) are too large to be tractable. However, since transposition does not change the eigenvalues, we can use the transpose to efficiently compute these eigenvalues (see Appendix A). These raw eigenvalues (feature eigs in Table 3), most directly test the idea that eigenvalues alone are useful functional connectivity features.

**Table 3:** Feature groupings for summarization. eigs, non-RMT eigenfeatures and their combinations with other non-RMT eigenfeatures. rmt, RMT eigenfeatures and their combinations. tseries, baseline timeseries reductions.

Coarse grouping	Fine grouping	Feature id
eigs	eigs	eigs
	eigs max	eigsminmax10
		eigsminmax20
		eigsminmax5
	eigs middle	eigsmiddle10
		eigsmiddle20
		eigsmiddle40
	eigs smooth	eigs + eigs_smooth
		eigs + savgol
		eigs_savgol
		eigs_smooth
rmt	rmt + eigs	eigs + levelvar
		eigs + rigidity
		eigs + rigidity + levelvar
		eigs + unfolded
		eigs + unfolded + levelvar
		eigs + unfolded + rigidity
	rmt only	levelvar
		rigidity
		rigidity + levelvar
		unfolded
		unfolded + levelvar
		unfolded + rigidity
		unfolded + rigidity + levelvar
tseries	Location	T-max
		T-mean
		T-med
		T-min
		T-p05
		T-p95
	Scale	T-iqr
		T-rng
		T-rrng
		T-std

However, not all eigenvalues may have predictive utility. We also examine as features the central eigenvalues (defined as the middle 10%, 20%, or 40% of the sorted eigenvalues, eigsmiddle in Table 3) and tail eigenvalues (defined as the first and last 5%, 10%, and 20% of each tail of the spectrum, eigsminmax in Table 3).

#### 3.2.2 RMT features

To compare the observed eigenvalue distribution to some RMT theoretical predictions, the eigenvalues must be unfolded.^1^^,^^2^ The details and motivation behind the unfolding procedure are documented well elsewhere.^2^ Practically, the unfolding process can be viewed as a smoothing and rescaling procedure where the originally observed spectrum $\lambda_{1}\leq \ldots \leq \lambda_{n}$ is smoothly mapped to an unfolded spectrum $e_{1},\ldots ,e_{n}$, such that $\sum d_{i}/n\approx 1$, if $d_{i}=e_{i+1}-e_{i}$. For most empirical data, the true smoothing function is not known, and so must be approximated.^1^^,^^2^ Typically, a polynomial is chosen.^45^

Given empirically observed eigenvalues Λ, the spectral rigidity $\Delta_{3}(L)$ is calculated for any positive real value L<max(Λ) as

$$\Delta_{3}(L)={\left\langle \underset{A,B}{\min}\;\frac{1}{L}\int_{c}^{c+L}{\left(\eta (\lambda )-A\lambda -B\right)}^{2}\right\rangle }_{c},$$ (1)

where η(λ) is the number of unfolded eigenvalues less than or equal to λ, ${\left\langle \cdot \right\rangle }_{c}$ denotes the average with respect to all starting points c, and where A and B denote the slope and intercept, respectively, of the least squares fit of a straight line to η(λ) on [c,c+L].^2^ Viewing the unfolded eigenvalues as a timeseries, the spectral rigidity for a value L is the “average non-linearity” of all intervals of length L over the series.

The level number variance $\Sigma^{2}(L)$ or level variance, for short, is closely related to the spectral rigidity^1^ and is calculated as

$$\Sigma^{2}(L)={\left\langle \eta^{2}(L,c)\right\rangle }_{c}-{\left\langle \eta (L,c)\right\rangle }_{c}^{2},$$ (2)

where η(L,c) is the number of unfolded eigenvalues in [c,c+L], and where c, L, and ${\left\langle \cdot \right\rangle }_{c}$ are as above.^2^ Viewing the unfolded eigenvalues as an irregular timeseries, the level number variance is the variation of the number of samples in all intervals of length L over the series.

As eigenfeatures, we compute the unfolded eigenvalues, and the spectral rigidity and level number variance for all L∈{1,2,…,20}. These are unfolded, rigidity, and levelvar, respectively, in Table 3.

*Trimming variants*. As the unfolding procedure operates on the sorted eigenvalues and involves fitting a smooth polynomial or exponential to these values, extreme values are often omitted from fitting.^48^^,^^49^ The number of eigenvalues to trim is subjective, and unfortunately, with a large number of different source matrices, it is too destructive to naively use the same hard criterion (e.g., largest 10%, largest three) for all unfoldings.

We develop and test four trimming variants: no trimming, precision-based, largest, and middle, with less subjective criteria for determining trimming thresholds. The details of these trimming procedures are straightforward and can be found in Appendix B, or the source code.

*Unfolding variants*. Following any trimming, the eigenvalues are fit with a polynomial. Because the choice of degree has been known to dramatically impact certain analyses involving the spectral rigidity or level variance,^45^^–^^49^ we compute the rigidity and level variance with all possible combinations of our trimming procedures and unfolding polynomial degrees of 3, 5, 7, and 9.

#### 3.2.3 Smoothed eigenfeatures

When computing RMT eigenfeatures, the polynomial fitting during unfolding has a smoothing effect, as do the additional averaging ${\left\langle \right\rangle }_{c}$ operations. However, it is possible other, simpler transformations of the eigenvalues (e.g., uniform or Savitsky–Golay smoothing) might have equal or greater predictive utility. We thus also test smoothed variants of the eigenvalues as predictive features: the sorted eigenvalues are smoothed with either a uniform (moving average) filter or Savitsky–Golay, using window sizes of 3, 5, 7, and 9 to yield 8 total features (eigs_smooth and eigs_savgol in Table 3).

#### 3.2.4 Feature combinations

A combination of RMT and non-RMT eigenfeatures could be more predictive than either alone. We test this with a variety of eigenfeature combinations, with a focus on combinations that involve the simplest features (e.g., raw eigenvalues and unfolded eigenvalues) and then features involving additional processing (e.g., smoothed eigenvalues, level variance, and rigidity). Combined features are formed by concatenation so that if we have features $f_{1},\ldots ,f_{n}$ with dimensions $p_{1},\ldots ,p_{n}$, then the combined feature is $\left[f_{1};\ldots ;f_{n}\right]$ with $\sum p_{i}$ dimensions. The final combinations chosen can be found in Table 3, where the “+” symbol indicates concatenation.

#### 3.2.5 Slicing variants

The largest, middle, or smallest eigenvalues could be most useful in characterizing any given system. For example, if the smallest eigenvalues correspond to random/noise aspects of a system but differences in the nature of the system noise most differentiate between systems, then the smallest eigenvalues may have the most predictive utility. Likewise, the L value in each RMT eigenfeature defines the degree of locality in which we summarize the spectrum: with small L-values, the spectral rigidity summarizes the non-linearity of eigenvalues that are relatively close to each other in magnitude. At large values of L, the rigidity summarizes the long-range non-linearity of the spectrum.

Since predictive utility may vary with summary locality or with the region of the original spectrum, we investigate various front, middle, and end contiguous slices of each eigenfeature in all analyses, where the size of each slice is either the first or last 5%, 10%, or 20% of the full eigenfeatures for non-middle slices, or the middle 10%, 20%, or 40% of the full eigenfeature for the middle eigenvalues.

For combined features, slicing variants are also computed, with slicing performed first on each feature to be combined. For example, if we have features $f_{1},\ldots ,f_{n}$ with dimensions $p_{1},\ldots ,p_{n}$, then the sliced max 25% feature for each component feature, ${\hat{f}}_{i}$ is the last $p_{i}/4$ elements of $f_{i}$, and the combined sliced feature is [f^1;…;f^n].

#### 3.2.6 Baseline features

If RMT or eigenvalue-based features fail to predictively outperform features that are simple summary statistics of the fMRI data, it is difficult to justify the greater computational and interpretational complexities of the former. We compute 10 simple statistical reductions of the voxel time series (“tseries” features in Table 3) to help assess the relative value of RMT features. That is, each statistic reduces the fMRI data along the voxel dimension, yielding a feature of t dimensions (e.g., the “mean” feature, T-mean, is the usual global mean signal).

The baseline reductions used as statistical summaries were: robust measures of location (mean, max, and min); non-robust measures of location (median, 95th percentile, and 5th percentile); non-robust measures of scale (standard deviation and the range—T-rng in Table 3); and robust measures of scale (interquartile range and difference between 95th percentile and 5th percentile—T-rrng in Table 3).

We do not take slice variants of these baseline features since the baseline features are still time series. However, we do also evaluate a number of smoothing degrees of these features, to account for noise and to be somewhat similar to the various smoothing variants for the eigenfeatures. Each baseline feature is tested with a degree of uniform smoothing, where the size of the smoothing window is either 1 (no smoothing), 2, 4, 8, or 16.

### 3.3 Classifiers

We use a variety of standard machine-learning classifiers available in the Scikit-learn^74^ Python library to solve each classification task. We use a gradient-boosted decision tree, random forest classifier, support vector classifier (SVC) with radial basis function, and k-nearest neighbors classifiers with k equal to 3, 5, and 9 (KNN3, KNN5, and KNN9, respectively), in all cases with the default hyperparameter values. We originally also attempted to test a simple logistic regression classifier but found that this model frequently failed to converge for a number of features, even after increasing iterations significantly.

### 3.4 Preprocessing Levels

Each preprocessing intermediate in the (1) brain extraction, (2) slice time correction, (3) motion correction, and (4) template registration pipeline was saved and used for entirely separate feature analyses. For example, all features were extracted from an fMRI image prepared with one of four levels or degrees of preprocessing, where preprocessing level k includes preprocessing steps 1 to k, inclusive, and starting at k=1.

### 3.5 Normalization

Because of the exponential distribution of the eigenfeatures, normalization is somewhat non-trivial, and a simple standardization or min–max normalization is ineffective. We instead first apply a logarithm to all eigenfeatures and then test each classifier with un-normalized or min–max normalized versions of the log-features. Baseline features are also tested with un-normalized and min–max normalized versions, but without any log transform.

### 3.6 Multiverse Analysis

We perform a “multiverse analysis^75^” to assess the overall predictive potential of the various eigenfeatures across all previously mentioned analytic choices. That is, for each combination of comparison task, classifier, and analytic choices, we use fivefold cross validation and use the mean area under the receiver operating characteristic curve (mAUROC) across folds as our metric to evaluate feature predictive utility.

The area under the receiver operating characteristic curve (AUROC) metric was chosen because it handles class imbalances present in the various datasets and is naturally normalized and interpretable such that mAUROC<0.5 indicates predictive performance worse than guessing, and mAUROC>0.5 indicates positive predictive utility.^76^ However, we also collected as performance metrics the accuracy, adjusted accuracy (proportion of samples in the entire dataset largest class minus accuracy), and the F1 score. A complete table with these additional metrics is available with the data for this study.

Each non-baseline feature is thus evaluated, for each dataset classification task and each classifier, exactly 1280 times: there are 4 preprocessing levels × 2 normalization methods × 4 trimming choices × 4 unfolding degrees × 10 slice choices. Each baseline feature is evaluated exactly 40 times for each comparison task and classifier: 4 preprocessing levels × 2 normalization methods × 5 smoothing window sizes. In total, with all the datasets and features examined in this study, this yields 2,053,920 mAUROC values to summarize, with five main analytic factors—preprocessing, normalization, trimming, unfolding/smoothing degree, and slicing—to consider.

### 3.7 Software Release

Because the computation of these statistics is non-trivial and to make the unfolding procedure more transparent and reproducible, we developed and release a separate, open-source Python library empyricalRMT.^77^ The library allows for efficient, parallel computation of the spectral rigidity and level variance via Monte Carlo methods, and automatically ensures convergence of the statistics to user-specified tolerances. The library also makes available various other functions useful for empirical RMT analyses, such as unfolding and trimming functions, and plotting facilities for classic RMT ensembles.

## 4 Results

### 4.1 Overview

Summarizing this quantity of data requires some caution. Measures of location or scale, even when robust, are, for the most part, misleading and uninformative when distributions are skewed. In most cases, we find skewed mAUROC distributions, and so have chosen primarily to present our findings visually, with kernel density estimates. Because aggregating across datasets with different class imbalances can be extremely misleading, even with a metric like the mAUROC,^78^ we restrict such summaries to Table 4 only, and note that Table S1 in the Supplementary Material is intended as a supplement only to the distribution plots: no conclusions should be drawn about feature performance from the table values alone.

**Table 4:** Numerical summaries of feature mAUROCs across predictable comparisons, and all combinations of analytic choices, sorted by 95% percentile (robust max) value. Bold values indicate column “best” values, when reasonable.

Feature	Mean	Min	5%	50%	95%	max	std
eigs + eigs_smooth	**0.583**	0.188	0.408	0.567	**0.788**	0.906	0.111
eigs + savgol	0.580	0.188	0.408	**0.563**	0.785	0.913	0.110
Unfolded	0.568	0.150	0.400	0.555	0.775	0.931	0.108
Unfolded + levelvar	0.569	0.150	0.400	0.555	0.775	0.925	0.108
Unfolded + rigidity	0.568	0.150	0.401	0.555	0.775	0.925	0.108
Unfolded + rigidity + levelvar	0.568	0.150	0.401	0.555	0.775	0.931	0.108
eigs + rigidity + levelvar	0.564	0.202	0.401	0.548	0.763	**0.937**	0.105
eigs + unfolded	0.564	0.202	0.401	0.549	0.763	0.919	0.105
eigs + unfolded + levelvar	0.564	0.202	0.400	0.548	0.763	0.933	0.105
eigs + unfolded + rigidity	0.564	0.202	0.401	0.548	0.763	0.920	0.105
eigs + levelvar	0.563	0.202	0.400	0.548	0.758	0.914	0.104
eigs	0.563	0.202	0.401	0.548	0.756	0.875	0.104
eigs + rigidity	0.563	0.202	0.400	0.548	0.756	0.882	0.104
eigs_smooth	0.569	0.188	0.417	0.556	0.755	0.907	0.102
T-p05	0.526	0.249	0.340	0.500	0.754	0.856	0.110
eigs_savgol	0.566	0.188	0.410	0.553	0.748	0.909	0.102
eigsminmax20	0.556	0.245	0.411	0.544	0.738	0.906	0.098
eigsminmax10	0.550	0.231	0.409	0.538	0.737	0.906	0.095
eigsminmax5	0.549	0.213	0.400	0.539	0.729	0.888	0.096
T-mean	0.557	0.208	**0.427**	0.558	0.717	0.814	0.085
levelvar	0.534	0.075	0.367	0.527	0.712	0.879	0.101
eigsmiddle40	0.547	**0.324**	0.419	0.533	0.697	0.785	0.084
Rigidity + levelvar	0.529	0.154	0.377	0.523	0.696	0.888	0.094
Rigidity	0.529	0.154	0.378	0.523	0.694	0.888	0.094
T-rrng	0.545	0.281	0.384	0.542	0.688	0.759	0.090
eigsmiddle20	0.545	0.321	0.419	0.533	0.686	0.798	0.080
T-iqr	0.494	0.175	0.312	0.490	0.682	0.759	0.099
eigsmiddle10	0.541	0.323	0.411	0.530	0.681	0.755	0.081
T-std	0.513	0.252	0.392	0.500	0.679	0.729	0.086
T-rng	0.538	0.279	0.392	0.539	0.678	0.779	0.092
T-max	0.540	0.275	0.385	0.546	0.678	0.754	0.095
T-med	0.539	0.221	0.399	0.541	0.665	0.702	0.077
T-p95	0.524	0.294	0.402	0.514	0.653	0.693	0.077
T-min	0.504	0.321	0.426	0.500	0.582	0.698	0.046

In addition, a large number of interactions are possible between each analytic factor in this study. For example, it could be that trimming impacts the overall mAUROC distribution only at a certain preprocessing level and for certain feature slicing. However, as there are too many potential interactions to present, we limit our presentation to main effects.

To aid in summarizing the performances of the large number of different features, we also summarize patterns of results across two abstract groupings of similar features (“coarse” and “fine”) shown in Table 3. Thus, for example, figures depicting the coarse feature grouping “eigs” in fact depict all observed mAUROC values for the fine feature groupings of raw, max, middle, and smoothed eigenvalues, and the fine feature grouping “eigs smooth,” for example, depicts all observed mAUROC values for both the eigs_savgol and eigs_smooth features.

### 4.2 Classification Tasks

#### 4.2.1 Non-predictable comparisons

It is important, to reduce figure clutter and complexity, to exclude a classification task or an analytic factor from summaries if there is no evidence that the classification task is solvable in general, or if, when restricting the analytic factor to a particular instance, there is no evidence of solvability. For example, if a particular trimming procedure were to render all classification tasks unsolvable, it would be better to note this and exclude the associated mAUROCs from further visualizations, rather than to have the mAUROC distributions diluted by the bad procedure.

**Fig. 1:**
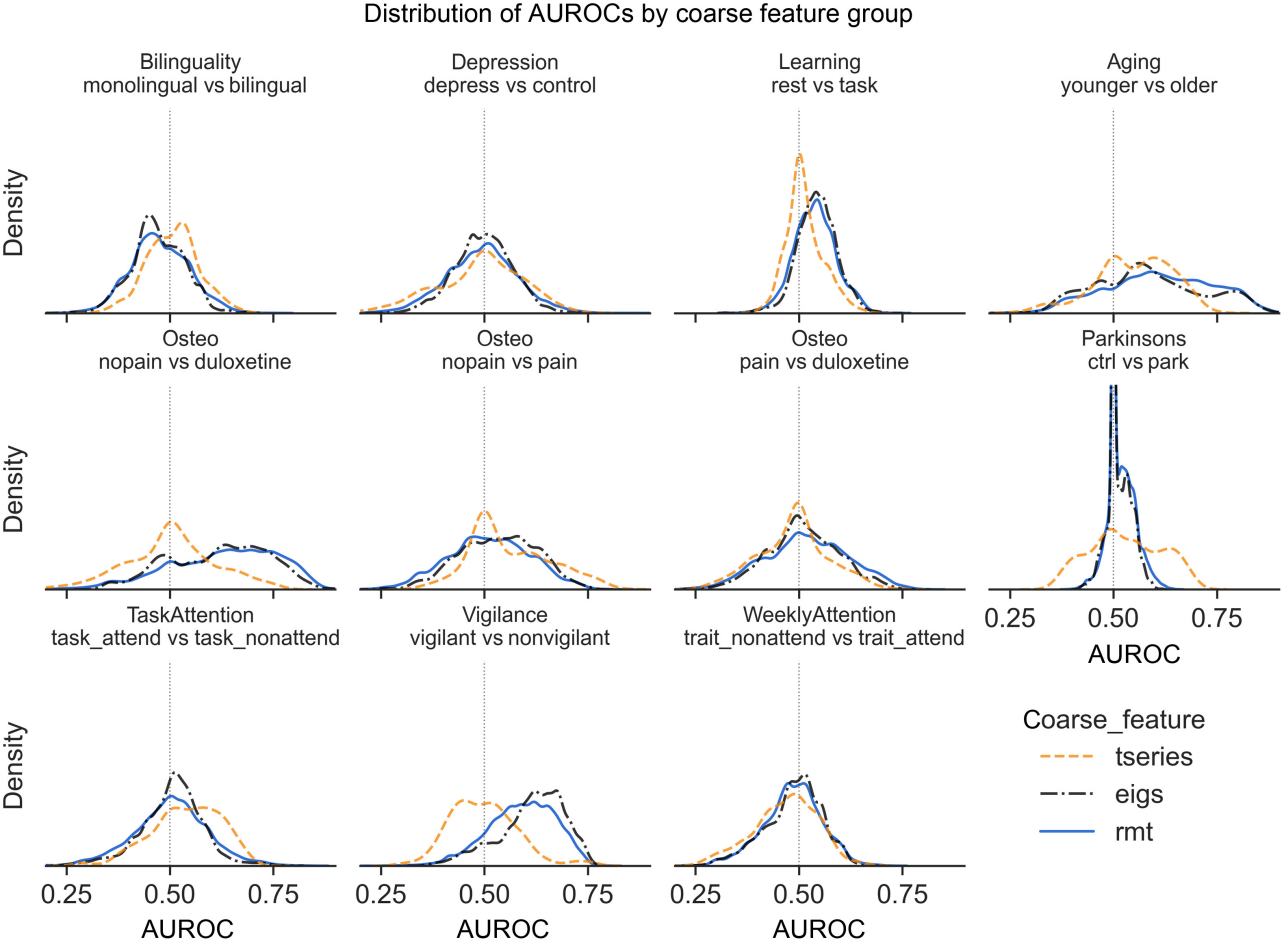
AUROC distributions across gross feature groupings and comparison tasks.

We take as lack of evidence of solvability an mAUROC distribution that is either (1) roughly symmetric and has mean and median close to 0.5, i.e., performance appears random or (2) with median and mode <0.5. Based on the mAUROC distributions for either coarsely grouped (Fig. 1) or finely grouped features (Fig. S1 in the Supplementary Material), neither bilinguality nor depression was predictable by either baseline or eigenfeatures. Additionally, the pain v duloxetine comparison in the osteo dataset, and the trait_attend v trait_nonattend conditions (“WeeklyAttend” in Fig. 1 and Fig. S1 in the Supplementary Material) in the “attention” dataset were also not meaningfully predictable by any feature. As such, we exclude these classification tasks from further figures and discussion. We did not, however, find that any analytic factor resulted in unsolvability.

#### 4.2.2 Predictable comparisons

As visible in Fig. 1, when coarsely summarizing features, eigenfeatures were more likely to be predictively useful than not, and except for in the task_attend v task_nonattend comparison, were also more predictively useful than the baseline features. Eigenfeatures most strongly and consistently demonstrated predictive utility in the aging dataset older v younger classification task, the osteo dataset nopain v duloxetine task, and in the attention vigilant v nonvigilant comparison.

### 4.3 Largest mAUROCs

**Fig. 2:**
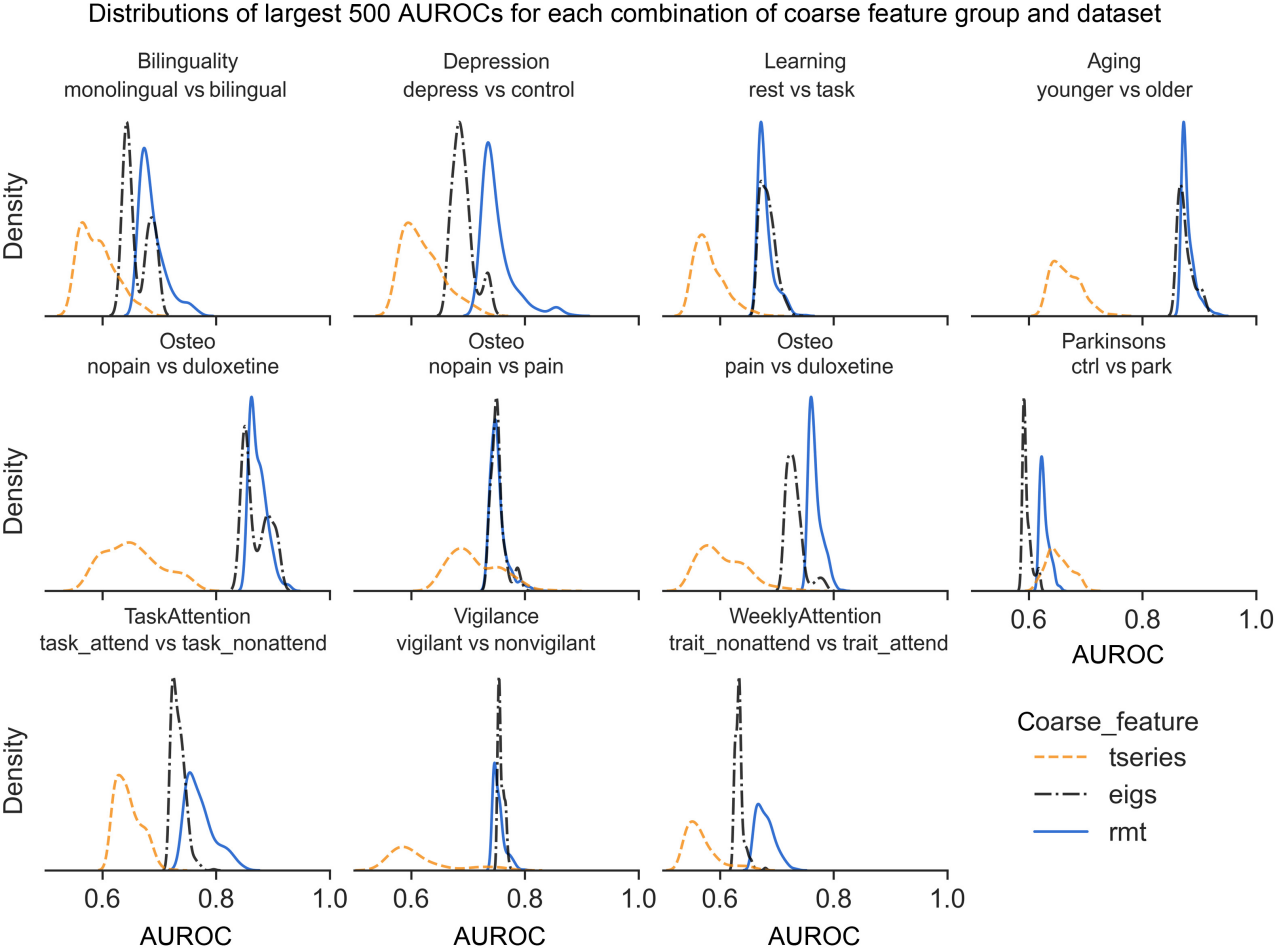
Distributions of largest 500 mAUROCs by coarse feature grouping.

Considering the various analytic choices as tunable parameters, it makes sense to examine the largest portion of mAUROCs as an indication of the maximum predictive potential of the eigenfeatures. In this case, it is clear that eigenfeatures using RMT features almost always had the highest potential predictive utility (Fig. 2). Figure S2 in the Supplementary Material shows that this was primarily due to either the “rmt only” or “rmt + eigs” features (see Table 3). However, RMT eigenfeatures were also most likely to cause poor performance and overfitting (indicated by an mAUROC<0.5; Fig. S3 in the Supplementary Material).

Examining these RMT features more closely, it is clear that these features’ performance distributions differ mostly due to the unfolding procedure. That is, combined features that used the unfolded eigenvalues plus some other RMT eigenfeature tended to have visually indistinguishable mAUROC distributions to those using the unfolded eigenvalues alone (Figs. S7 and S8 in the Supplementary Material). Instead, the mAUROC distributions of these features differed mostly in the tails (Figs. S2 and S3 in the Supplementary Material).

### 4.4 Effect of Preprocessing

**Fig. 3:**
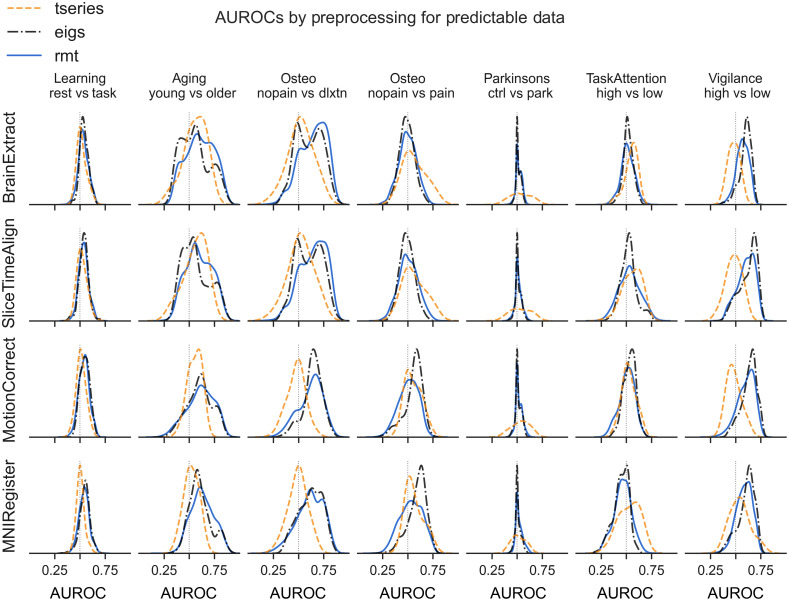
mAUROC distributions by preprocessing degree.

At a coarse level of feature grouping, slice time correction followed by motion correction tended to slightly increase the predictive utility of the eigenfeatures (Fig. 3) relative to brain extraction only. Subsequent registration following these steps did not generally impact mAUROC distributions further, except in the task_attend v task_nonattend comparison, where registration reduced the predictive utility of the eigenfeatures (Fig. 3, second-last column). When examining features more finely, it is clear that preprocessing most impacts the mAUROC distribution of the largest and central eigenvalues (“eigs middle” and “eigs max” in Fig. S4 in the Supplementary Material).

### 4.5 Effect of Classifier

Within any feature grouping (coarse or fine) and within a classification task, mAUROC distributions were generally similar across classifiers (Figs. S5 and S6 in the Supplementary Material). Additionally, these figures also show that within each classification task, choice of classifier does not result in dramatic changes to the rough rank ordering of features. For example, if features are ranked on predictive utility, using the median, modal, or bulk of the mAUROC values, this rank ordering appears to remain similar across classifiers.

In the attention task_attend v task_nonattend condition, eigenfeatures were modally predictive only with the RF classifier, whereas in the aging data, an SVC was least likely to have mAUROC<0.5. Overall, however, differences in the mAUROC distributions due to the classifier were small and inconsistent.

### 4.6 Normalization

There was no visible effect of feature normalization on mAUROC distributions.

### 4.7 Effect of Trimming

Trimming based on numerical precision (see Sec. 3.2.2 and Appendix B) did not result in meaningfully different mAUROC distributions in any case (Fig. S7 in the Supplementary Material). However, trimming away the largest, or both largest and smallest eigenvalues, generally had a significant positive effect on the predictive quality of RMT features, most especially for RMT features involving the unfolded eigenvalues. When employing these trimming methods, these features were consistently more predictive than not (Fig. S7 in the Supplementary Material).

### 4.8 Effect of Unfolding Degree

The choice of polynomial unfolding degree significantly impacted the mAUROC distributions for most classification tasks and most RMT features, and most significantly for the level variance features (Fig. S8 in the Supplementary Material). Overall, Fig. S8 in the Supplementary Material weakly suggests that either smaller (degree = 3) or larger (degree = 9) unfolding degrees tend to yield the most predictively useful RMT features. However, when restricting to the most predictive RMT features (those including the unfolded eigenvalues), it seems clear from Fig. S8 in the Supplementary Material that the largest unfolding degree of 9 produces the most favourable mAUROC distributions.

### 4.9 Effect of Slicing

The best slice size and location depended complexly on the classification task and feature, and few general summaries can be made of these interactions. However, features including the full spectrum (e.g., raw eigenvalues, smoothed eigenvalues, and their combinations with RMT eigenfeatures) were slightly more predictive when using the largest portions (“max-XX,” rows in Fig. S9 in the Supplementary Material), and usually least predictive when features primarily involved the smallest or central portions.

### 4.10 Choice of Summary Metric

We note briefly that most of the above findings regarding the impacts of analytic factors, and rank ordering of feature predictive utilities, are similar when using the adjusted accuracy (see Sec. 3.6), instead of the mAUROC (see Figs. S10–S18 in the Supplementary Material). However, if comparison task predictability is defined as requiring adjusted accuracies to be more positive than negative, then the learning and Parkinson’s datasets do not appear to be predictable with any feature (Fig. S10 in the Supplementary Material).

## 5 Discussion

In this study, eigenfeatures inspired by RMT and derived from the eigenvalues of the full, whole-brain voxelwise fMRI correlation matrix were found to have predictive utility across a wide variety of phenomena and analytic choices. Compared to simple baseline reductions of the fMRI data, these eigenfeatures had more consistent predictive utility and a higher maximum predictive potential (Figs. 1 and 2). In addition to evidence from previous studies,^29^^,^^31^^,^^32^ this suggests RMT may be a useful analytic and theoretical tool for understanding functional connectivity.

However, eigenfeature mAUROC values observed in this study were highly sensitive to the overall analytic procedure, and there was no single analytic choice (e.g., choice of trimming procedure, unfolding polynomial degree, number of preprocessing steps, or feature slicing) that ensured, for any combination of feature and classification task, that all other analytic choices resulted in mAUROC values >0.5. In addition, the mean, median, and modal mAUROCs were generally close to 0.5 and adjusted mean and median accuracies also tended to be close to zero. Thus we find limited evidence that functional-connectivity-based eigenfeatures have general, “out of the box” predictive utility, with general utility likely requiring either careful tuning, or different preprocessing decisions and analytic choices than those examined here.

Nevertheless, in all datasets, there were combinations of analytic choices that resulted in cross-validated mean prediction performances well beyond mere guessing (Figs. S2 and S11 in the Supplementary Material and Table 5). Whether or not these should be considered to have practical relevance depends on one’s goals, however, we note that with small datasets of rs- or task-fMRI data, binary, subject-level classification using whole-brain features is generally challenging.

**Table 5:** Top three robust maximum (95th percentile) mAUROC and adjusted accuracy (acc+) values for each predictable classification task and fine feature grouping, sorted by mAUROC. A dash indicates that the fine feature grouping for that row was not in the top three, i.e., that the top three performing features differed depending on the performance metric.

Classification task	Source feature	mAUROC	acc+
Aging: younger versus older	eigs smooth	0.834	0.226
	rmt + eigs	0.828	0.212
	eigs	0.823	0.211
Learning: rest versus task	rmt + eigs	0.638	0.007
	eigs	0.636	0.007
	eigs max	0.631	—
	eigs middle	—	0.012
Osteo: nopain versus duloxetine	rmt only	0.819	0.259
	eigs max	0.812	0.226
	rmt + eigs	0.806	—
	eigs smooth	—	0.212
Osteo: nopain versus pain	tseries loc	0.762	0.104
	tseries scale	0.697	—
	eigs smooth	0.684	—
	eigs middle	—	0.069
	eigs	—	0.034
Parkinsons: ctrl versus park	tseries scale	0.687	0.107
	tseries loc	0.657	0.063
	rmt only	0.590	0.027
TaskAttention: task_attend versus task_non-attend	tseries loc	0.666	0.135
	rmt only	0.660	0.121
	tseries scale	0.656	0.124
Vigilance: vigilant versus non-vigilant	eigs middle	0.733	0.184
	eigs smooth	0.726	—
	rmt + eigs	0.719	0.162
	eigs	—	0.162

For example, deep learning methods improve upon guessing by 17% for autism^79^ or 3% to 30% for ADHD,^80^ 16% for severe depression,^81^ and 23% for obsessive compulsive disorder.^82^ Manual feature engineering with more separable conditions (e.g., schizophrenia) can result in classification accuracies well above 90%,^83^ and with larger data, sophisticated custom feature extraction methods can achieve near perfect accuracies at classifying task versus rest.^84^ However, for functional connectivity data and with classical machine learning algorithms (such as SVC), we in general only expect large prediction accuracies (e.g., >80%) when the group functional connectivities are already strongly separated (e.g., Cohen’s d>1.0).^85^ In this study, the (robust) maximum improvements upon guessing are shown in Table 5 and vary from 3% to 26%.

It is somewhat surprising that the eigenfeatures examined here ever have net cross-validated predictive utility. The reduction of the functional connectivity matrix to the sorted t−1 eigenvalues uses all brain voxels (including gray matter voxels) and destroys a large amount of information (radically different matrices can have identical sorted eigenvalues). The subsequent small-degree polynomial fit used in the unfolding procedure further reduces variance in the raw data, and all eigenfeatures, due to the eigenvalue sorting, are monotonically increasing curves (or approximately monotonic). All such curves could likely be fit near-perfectly with 3 to 5 parameters, i.e., the inherent dimensionality of these features is quite small. Interpreting the eigenvalues as the magnitudes of the principal components of the standardized data, this suggests that a rough summary of the magnitudes of the principal components can often be surprisingly predictive in fMRI.

**Fig. 4:**
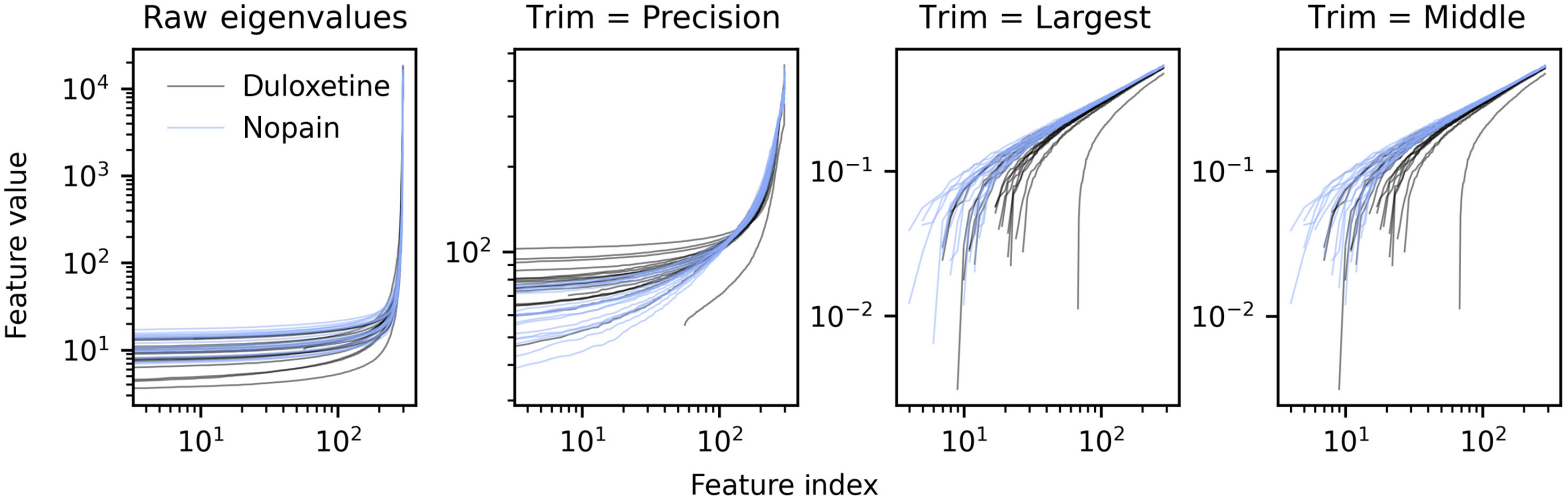
Raw eigenvalues (first subplot) and unfolded eigenvalues (polynomial degree 9; last 3 subplots) for osteo dataset duloxetine v nopain classification task, brain extraction, and slice time correction only. Median observed mAUROC = 0.643 (range = 0.346 to 0.925).

We speculate that the unfolded eigenvalues may have predictive utility in part because of their smoothing and rescaling effect (see also Sec. 4.3). Figure 4, which depicts the raw eigenvalues and unfolded eigenvalues with different trimming procedures, shows how the raw eigenvalues have strongly exponential distributions, even with logarithmic axes. This is due to the magnitude of the largest eigenvalues, and the unfolded and trimmed feature distribution in Fig. 4 are far less pathologically distributed.

### 5.1 Limitations

As it is unfortunately typical of fMRI research,^86^ the number of subjects in each dataset was quite small (Table 2). With such small numbers of subjects, randomization cannot be expected to effectively control group differences, and it is possible the predictive utility of the eigenfeatures was due to capitalization of such differences. For example, eigenfeatures were generally predictive in the aging dataset, and it is quite possible for randomization failures to introduce age imbalances across classes.

Likewise, one of the other more predictable classification tasks was the duloxetine v nopain task in the osteoarthritis dataset. An active drug could introduce any number of physiological confounds relevant to fMRI,^87^ but we could not control for such effects due to the absence of physiological recording in most datasets.

In general, much larger fMRI datasets are needed to adequately control for and test what exactly the connectivity eigenvalues actually predict and to test if these predictions generalize to larger, different populations.

## 6 Conclusion

Eigenvalue-based features inspired by RMT and extracted from fMRI functional connectivity matrices were found to have predictive utility across a wide variety of datasets and classification tasks. However, the predictive utilities were modest and highly dependent on preprocessing steps and other fitting and feature selection procedures.

Given the sensitivity to these decisions observed in this study and considering the lack of consensus regarding the preprocessing of fMRI or other complex biological signal data, RMT should probably not be currently considered a tool with “out-of-the-box” utility in these domains. Although RMT likely still has potential to yield insights in a variety of contexts, our results suggest that, absent strong evidence otherwise, it is not necessarily safe to assume that these insights will generalize broadly beyond the specific preprocessing and analytic pipelines involved.

Further research might establish specific sets of analytic choices that allow RMT to consistently extract useful information in a wide variety of contexts. However, it is also possible that each unique context might require a specific combination of choices. In this latter case, there should be strong theoretical justification for the use of RMT, and for each of the various analytic choices involved in its use: the variability of results seen in this study suggests that there may often be a set of analytic choices that provide favourable results, and which can be weakly justified by plausible, but ultimately *post hoc* justifications. Provided that these precautions are followed and that future studies employing RMT also carefully investigate the sensitivity of any findings to such analytic decisions, then there is likely considerable untapped potential for RMT in the analysis of fMRI.

## 7 Appendix A: Correlation Eigenvalues via Transposition

Let X be a real n×p matrix with n>p. Let

$$Z=\text{norm}(X)=(X_{1}-{\overset{¯}{X}}_{1}|\ldots |X_{p}-{\overset{¯}{X}}_{p}),$$

where $X_{i}$ denotes column i of X. Denote the (unordered) set of eigenvalues of X as eigs(X), and let $r={\left(p-1\right)}^{-1}$. Denote the covariance matrix of X as cov(X). Then:

$$\mathrm{eigs}(\mathrm{cov}(X))=\mathrm{eigs}(r\cdot {\mathrm{ZZ}}^{⊤})\;=r\cdot \mathrm{eigs}({\mathrm{ZZ}}^{⊤})\;=r\cdot \mathrm{eigs}({\left({\mathrm{ZZ}}^{⊤}\right)}^{⊤})\;=r\cdot \mathrm{eigs}(Z^{⊤}Z).$$

Denote the correlation matrix of X as corr(X), and let

$$Y=\text{standardize}(X)=((X_{1}-{\overset{¯}{X}}_{1})/\sigma_{1}|\ldots |(X_{p}-{\overset{¯}{X}}_{p})/\sigma_{p}).$$

Then

$$\mathrm{eigs}(\mathrm{corr}(X))=\mathrm{eigs}(\mathrm{cov}(Y))=r\cdot \mathrm{eigs}(Y^{⊤}Y).$$

## 8 Appendix B: Trimming Procedures

We implement three trimming procedures: precision-based, largest, and middle trimming. The source code is the definitive reference for the procedures, but we describe the motivations belows.

In precision-based trimming, we trim away any eigenvalues that are close enough to zero to be considered a result of numerical error due to floating point representation. There are two thresholds we consider, including the one used by NumPy^88^ for the determination of matrix rank, and those recommended by LAPACK^89^ in their user guide on the error bounds for symmetric eigenproblems and related additional details. We trim each matrix eigenvalues to whichever threshold is largest for the matrix in question.

For largest trimming, we must determine a threshold in which to separate “large” from “small” eigenvalues. The eigenvalues for our data tended to grow exponentially, so we instead looked at thresholding on the logarithms. We then used k-means with k=2 on the precision-trimmed eigenvalues and took the largest cluster (which also always had the smaller mean) as the “largest” eigenvalues to trim away. “middle” trimming simply reflects the threshold found by the largest trim method, e.g., if the largest trim method removes the last n precision-trimmed eigenvalues, then we also trim the first n smallest eigenvalues remaining after precision-trimming.

We chose one-dimensional k-means partly due to efficiency and simplicity and because of the general relation to classical thresholding methods such as the Otsu method.^90^

## Data, Materials, and Code Availability

All raw fMRI data used in this study are publicly available on the OpenNeuro platform.^52^^,^^91^ All codes used specifically to perform preprocessing and analyses for this paper is publicly available online in the paper GitHub repository.^92^ This repository also includes all produced prediction data in a single table available in the file all_produced_prediction_data.json.xz available in this paper repository. Code used to compute the spectral observables and perform unfolding is publicly available in the empyricalRMT library.^77^
