# Supplementary material for: Random matrix theory tools for the predictive analysis of functional magnetic resonance imaging examinations
Source: J Med Imaging (Bellingham). 2023 Jun 14;10(3):036003. doi: 10.1117/1.JMI.10.3.036003 (PMC10266090; doi:10.1117/1.JMI.10.3.036003)
Supplement: Supplementary file 1 [file JMI_010_036003_SD001.pdf]

Random Matrix Theory Tools for the Predictive Analysis of Functional Magnetic Resonance Imaging Examinations - Supplementary Material

Derek Berger[a], Gurpreet S. Matharoo[b,c], and Jacob Levman[a*,d,e]

[a]St. Francis Xavier University, Department of Computer Science, 4130 University Avenue, Antigonish, Nova Scotia, Canada, B2G 2W5
[b]St. Francis Xavier University, ACENET, 4130 University Avenue, Antigonish, Nova Scotia, Canada, B2G 2W5
[c]St. Francis Xavier University, Department of Physics, 4130 University Avenue, Antigonish, Nova Scotia, Canada, B2G 2W5
[d]Athinoula A. Martinos Center for Biomedical Imaging, 149 Thirteenth Street, Suite 2301, Charlestown, Massachusetts, United States, 02129
[e]Nova Scotia Health Authority, Research Affiliate, Nova Scotia, Canada

May 15, 2023

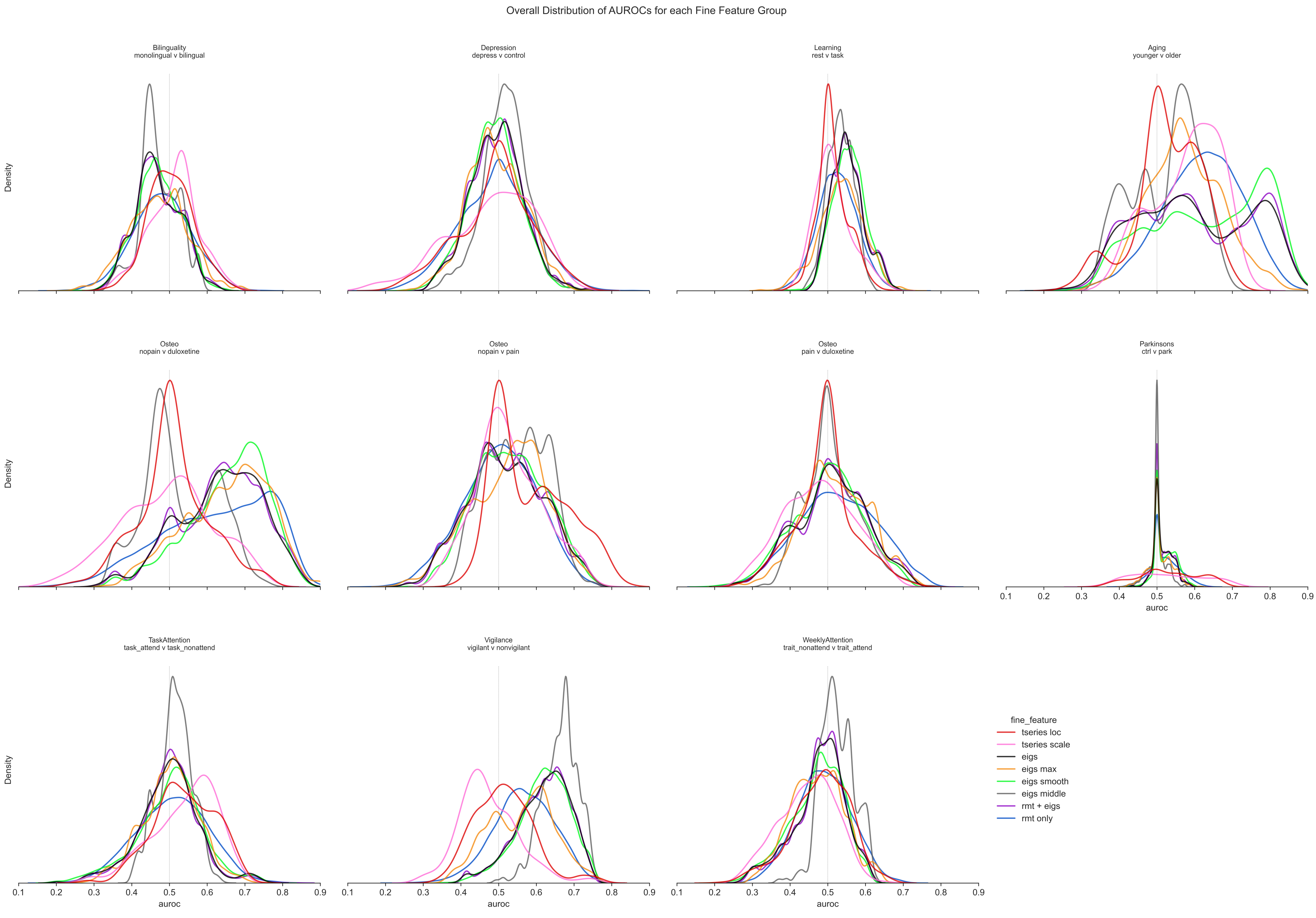

Figure 1: AUROC distributions across gross feature groupings and comparison tasks.

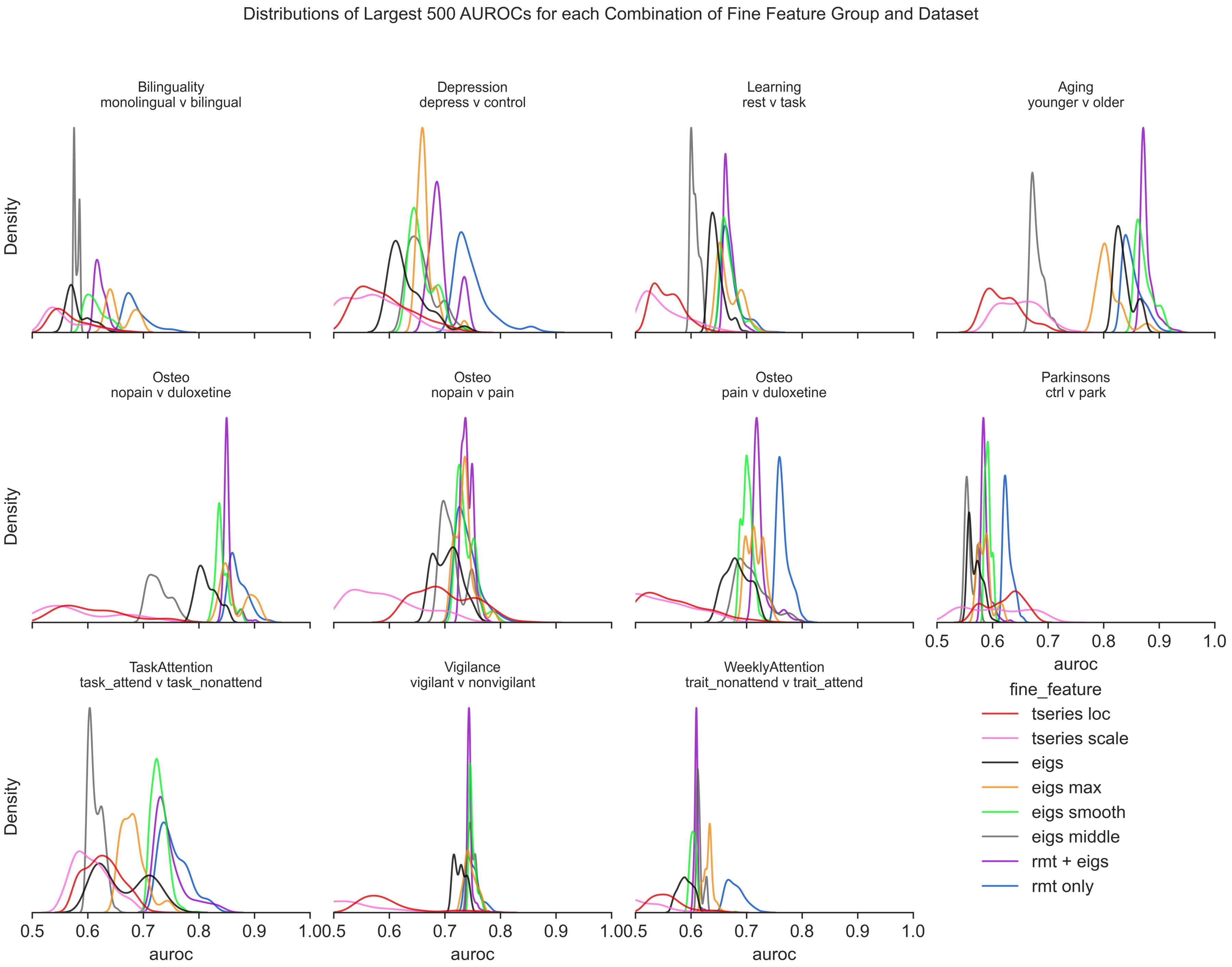

Figure 2: Distributions of largest 500 mAUROCs across fine feature grouping, by comparison task. Note “rmt only” and “rmt + eigs” features tend to have the best possible performances across predictable tasks.

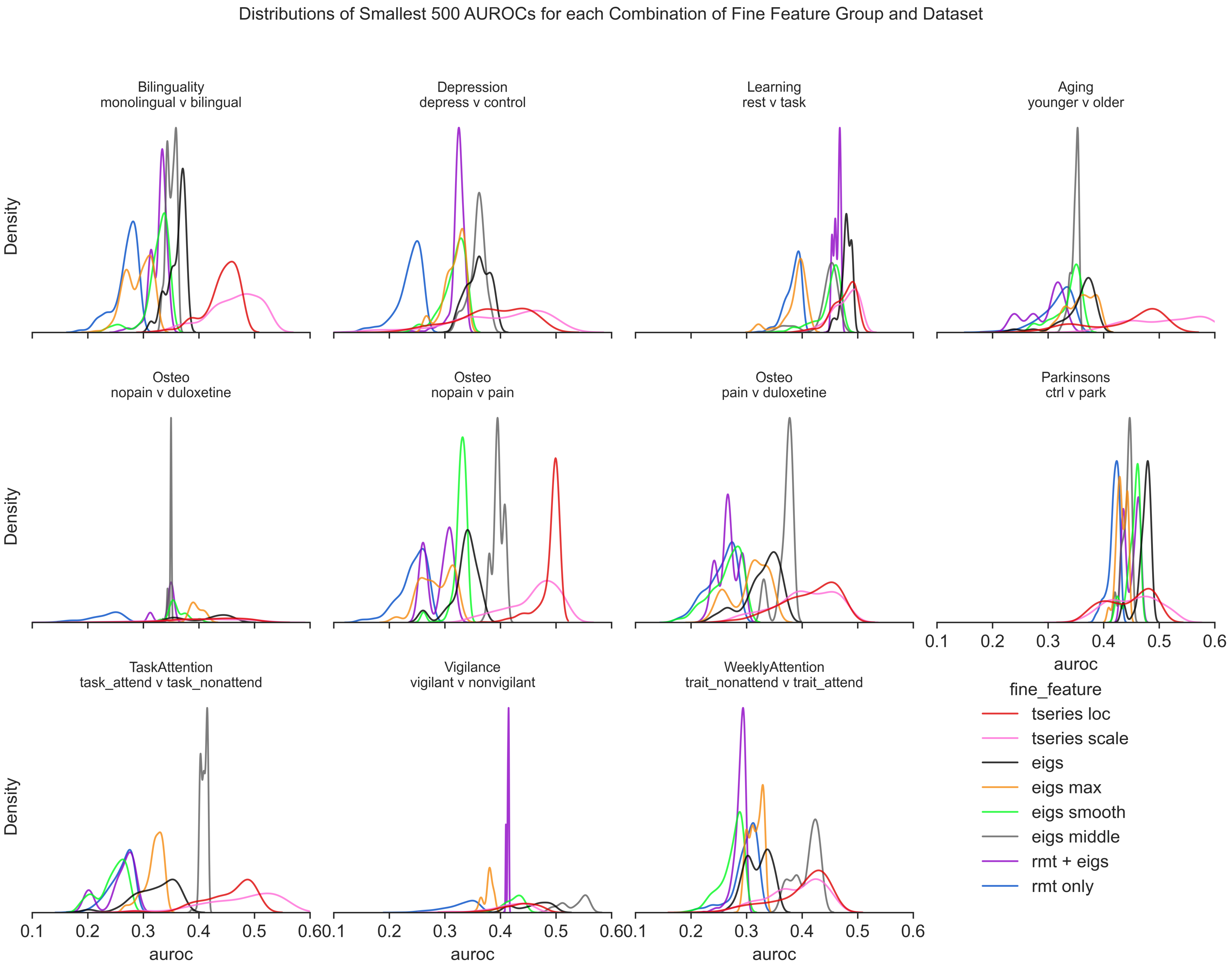

Figure 3: Distributions of smallest 500 mAUROCs across fine feature groupings, by comparison task. Note "rmt only" and "rmt + eigs" features tend to have the worse possible performances across predictable tasks.

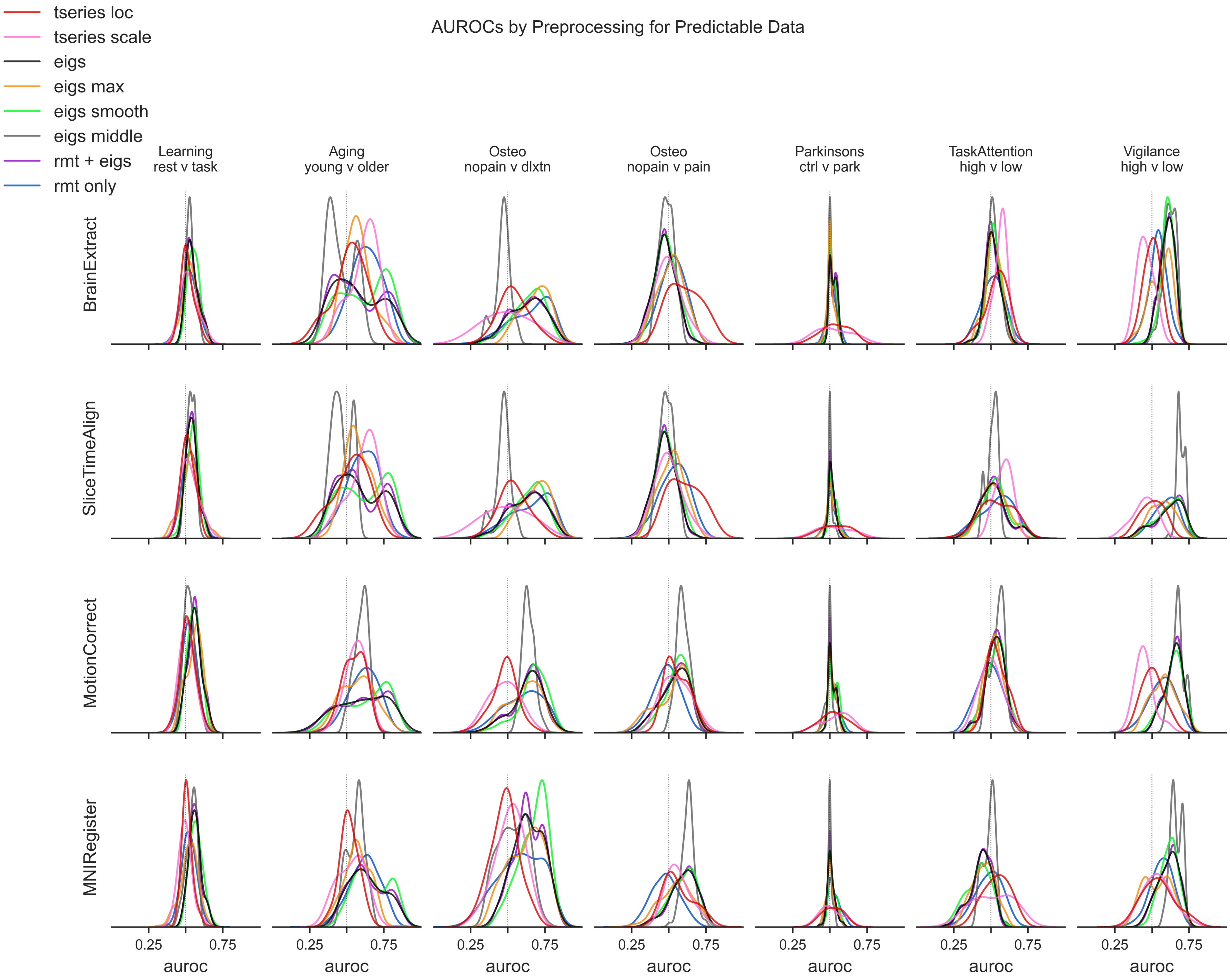

Figure 4: AUROC distributions across fine feature groupings and predictable comparison tasks, with effect of preprocessing.

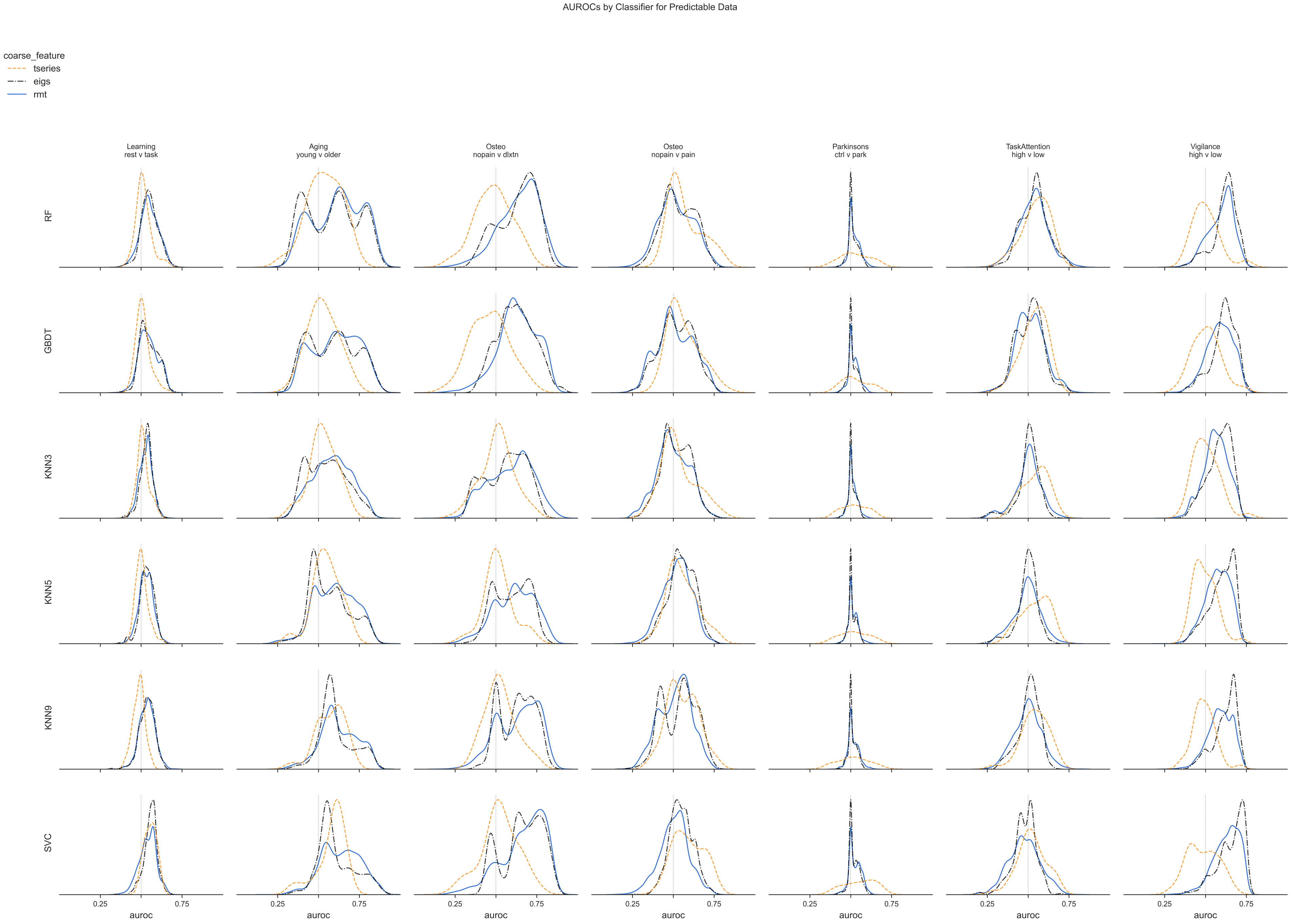

Figure 5: AUROC distributions across coarse feature groupings and predictable comparison tasks, by classifier. Note the general similarity of each distribution within a particular classification task (column) and within each feature grouping.

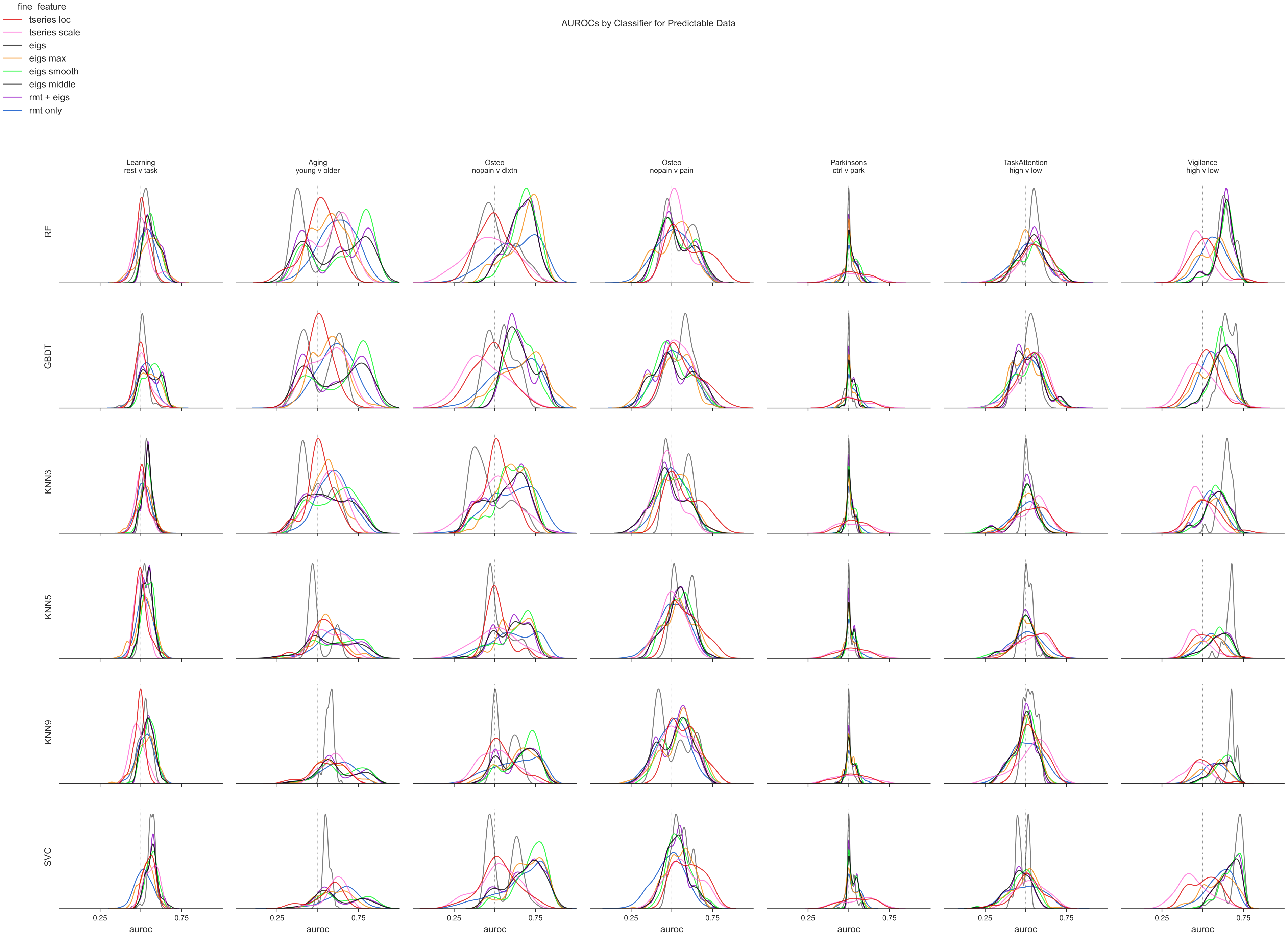

Figure 6: AUROC distributions across fine feature groupings and predictable comparison tasks, by classifier. Note the general similarity of each distribution within a particular classification task (column) and within each feature grouping. Note also that, within a classification task (column), that the rank ordering of features, based on wither the median, mode, or mean, does not change dramatically or consistently from classifier to classifier.

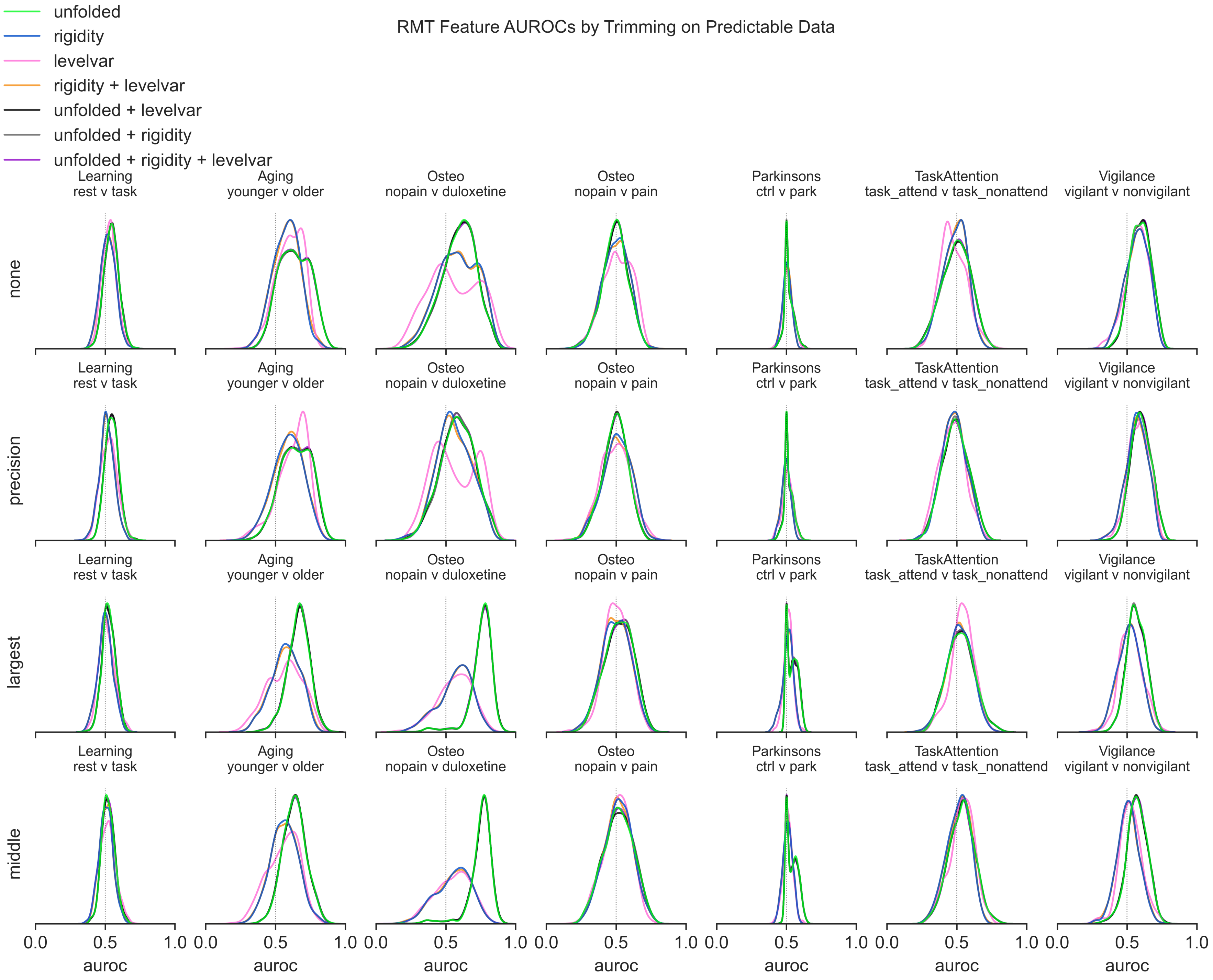

Figure 7: Distributions of mAUROCs for unfolding-dependent RMT features, by trimming. Note the tendency for a rightward shift in the mAUROC distributions of the features involving the unfolded eigenvalues when using largest or middle trimming (most dramatic in the Osteo nopain v duloxetine condition). The impact of these trimming methods on the rigidity and level variance features, however, was mixed (compare Vigilance data to Osteo nopain v duloxetine condition.)

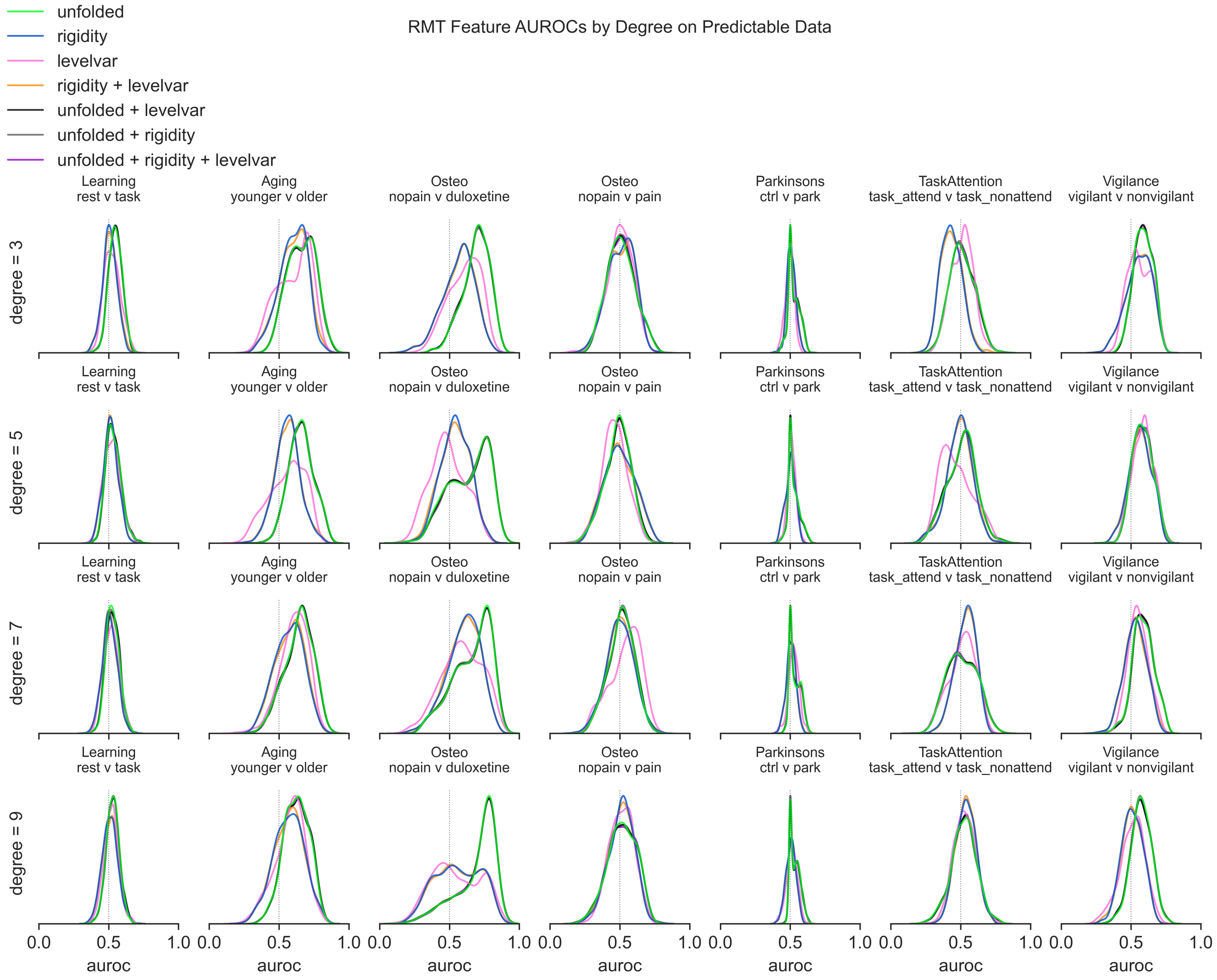

Figure 8: Distributions of mAUROCs for unfolding-dependent RMT features, by degree.

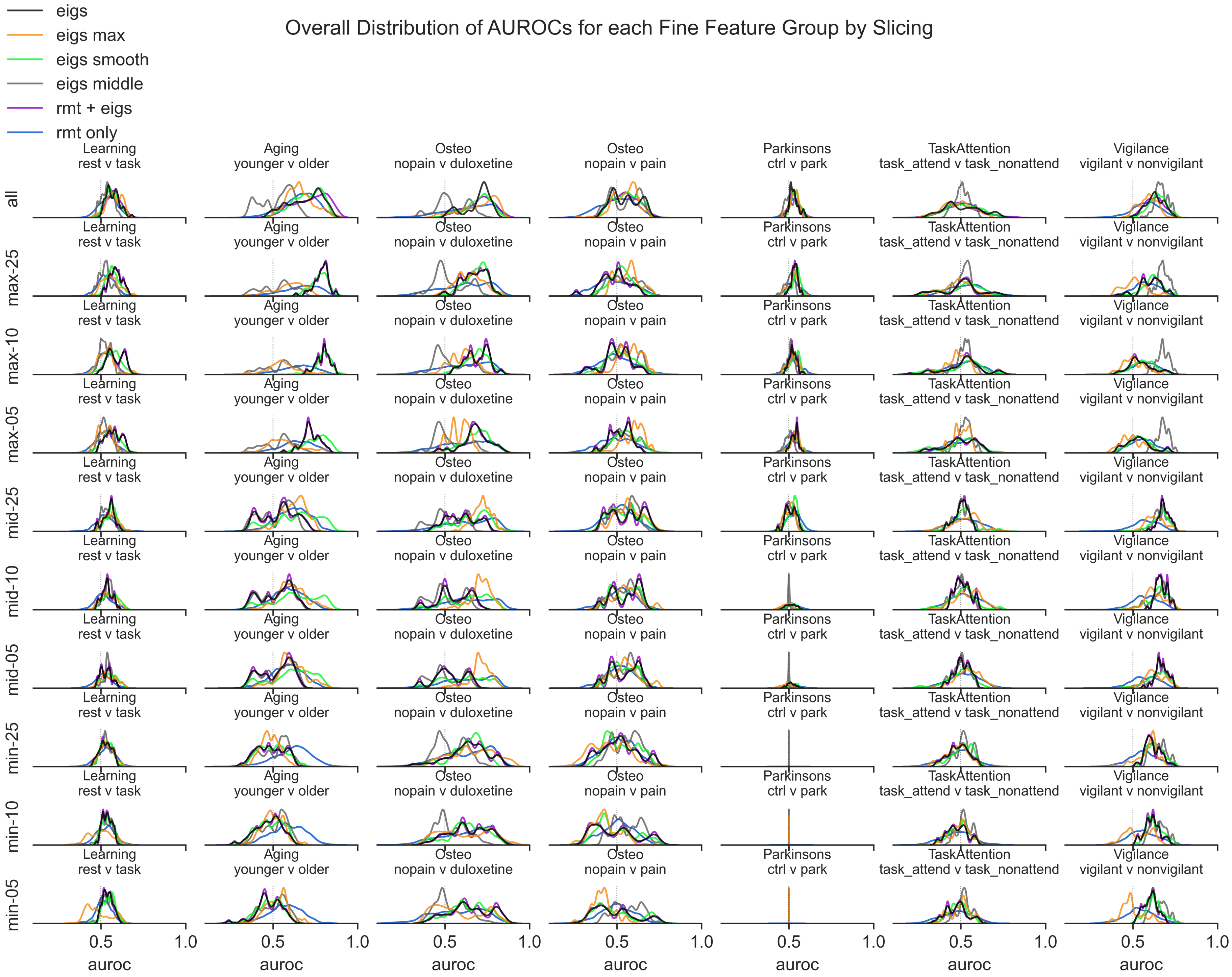

Figure 9: Distributions of mAUROCs by slicing. Features involving the full spectrum (raw eigenvalues, smoothed eigenvalues, and rmt + eigs) sometimes have most positive mAUROC distributions when using the larger eigenfeature values (first three columns) or middle values (Osteo nopain v pain condition, Vigilance classification task).

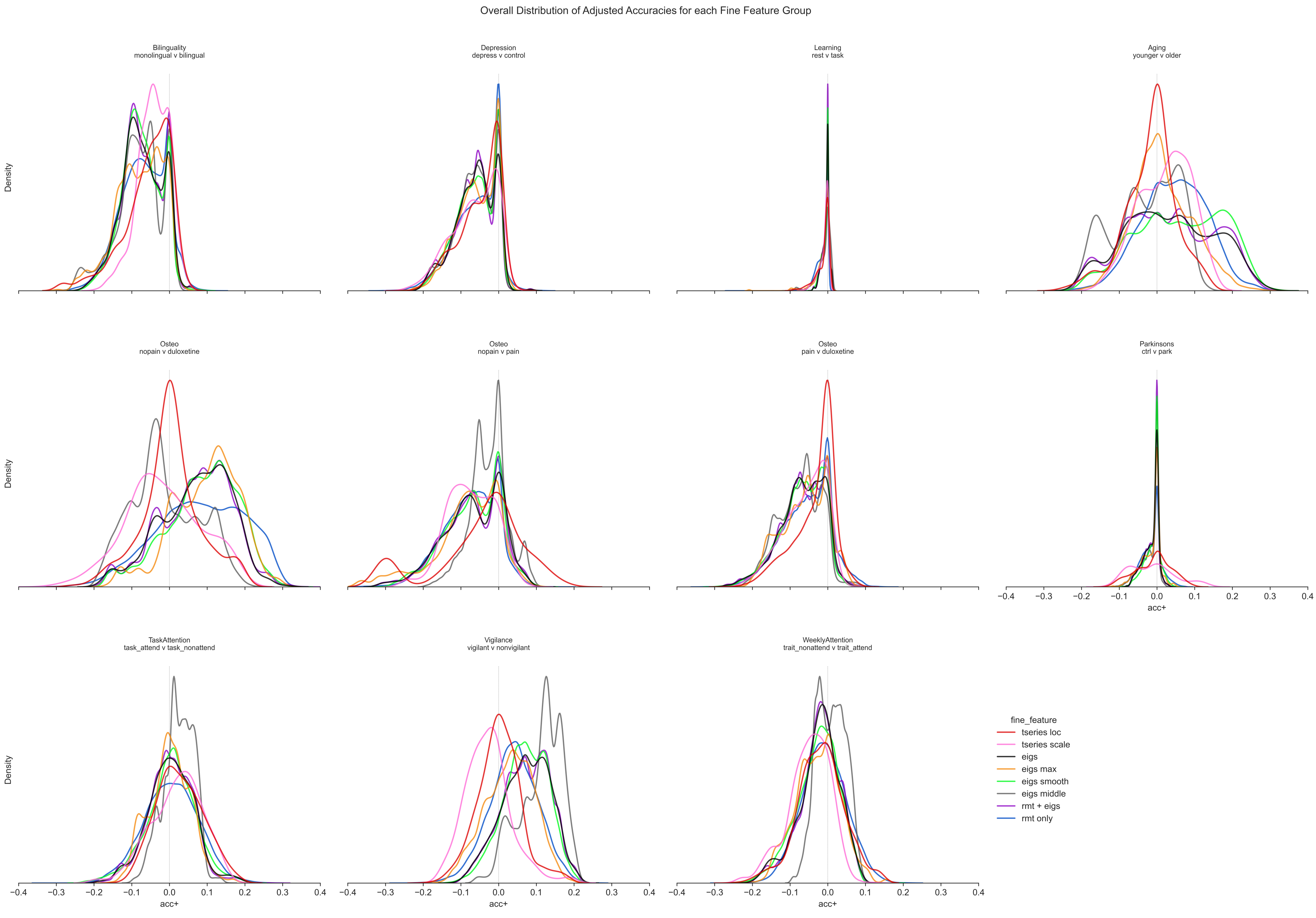

Figure 10: Adjusted accuracy distributions across fine feature groupings and comparison tasks.

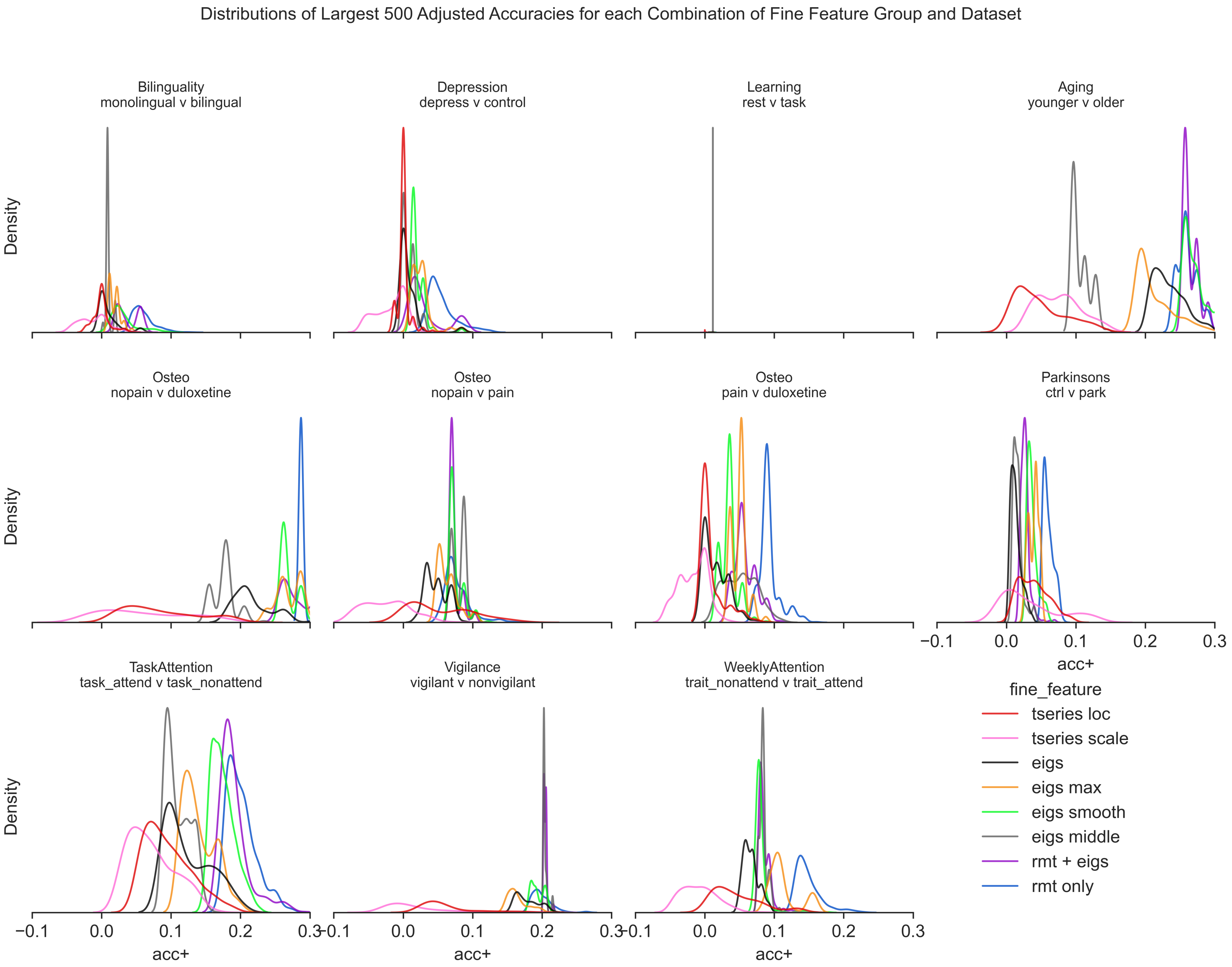

Figure 11: Distributions of largest 500 mean adjusted accuracies across fine feature grouping, by comparison task. Note “rmt only” and “rmt + eigs” features tend to have the best possible performances across predictable tasks.

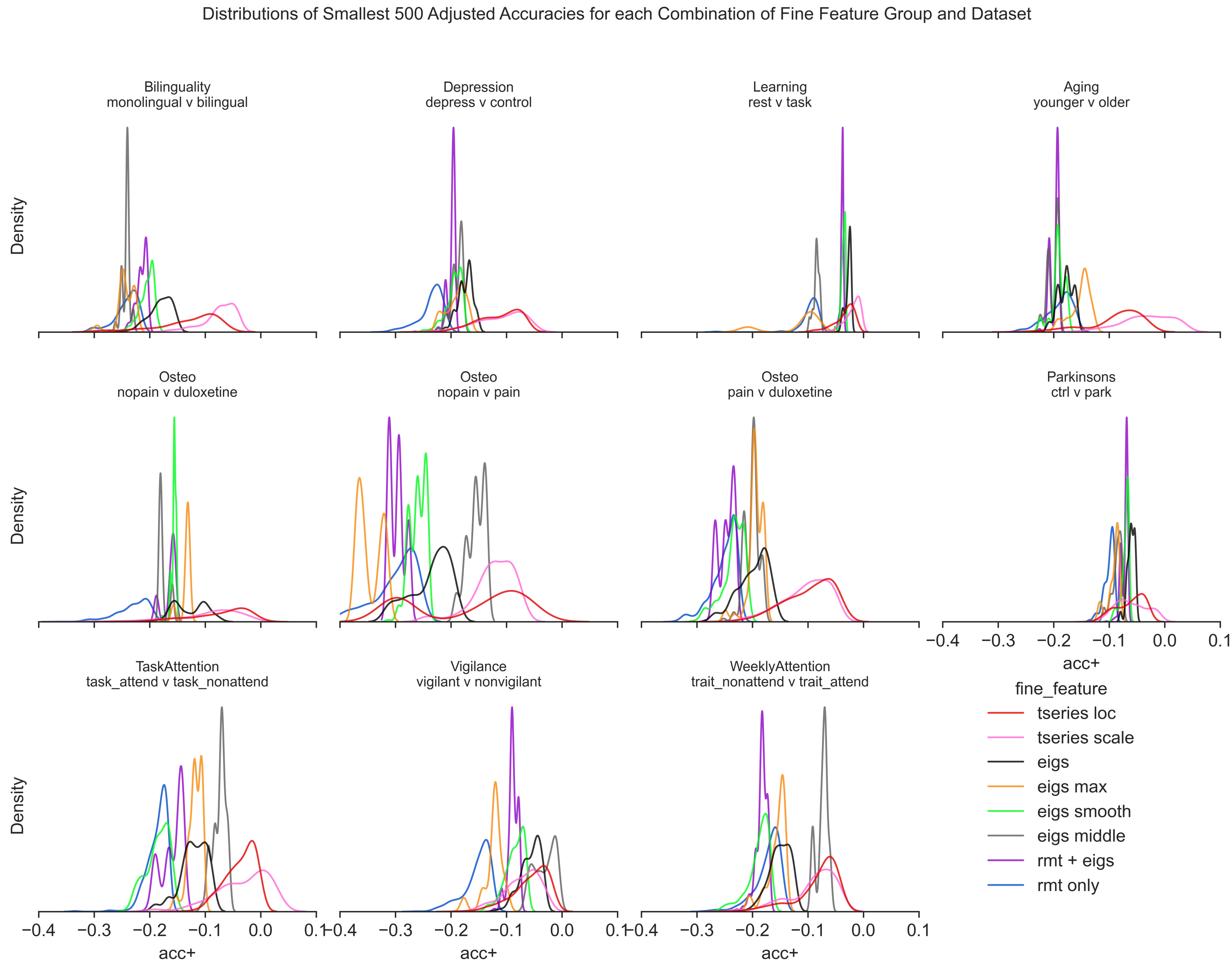

Figure 12: Distributions of smallest 500 mean adjusted accuracies across fine feature groupings, by comparison task. Note “rmt only” and “rmt + eigs” features tend to have the worse possible performances across predictable tasks.

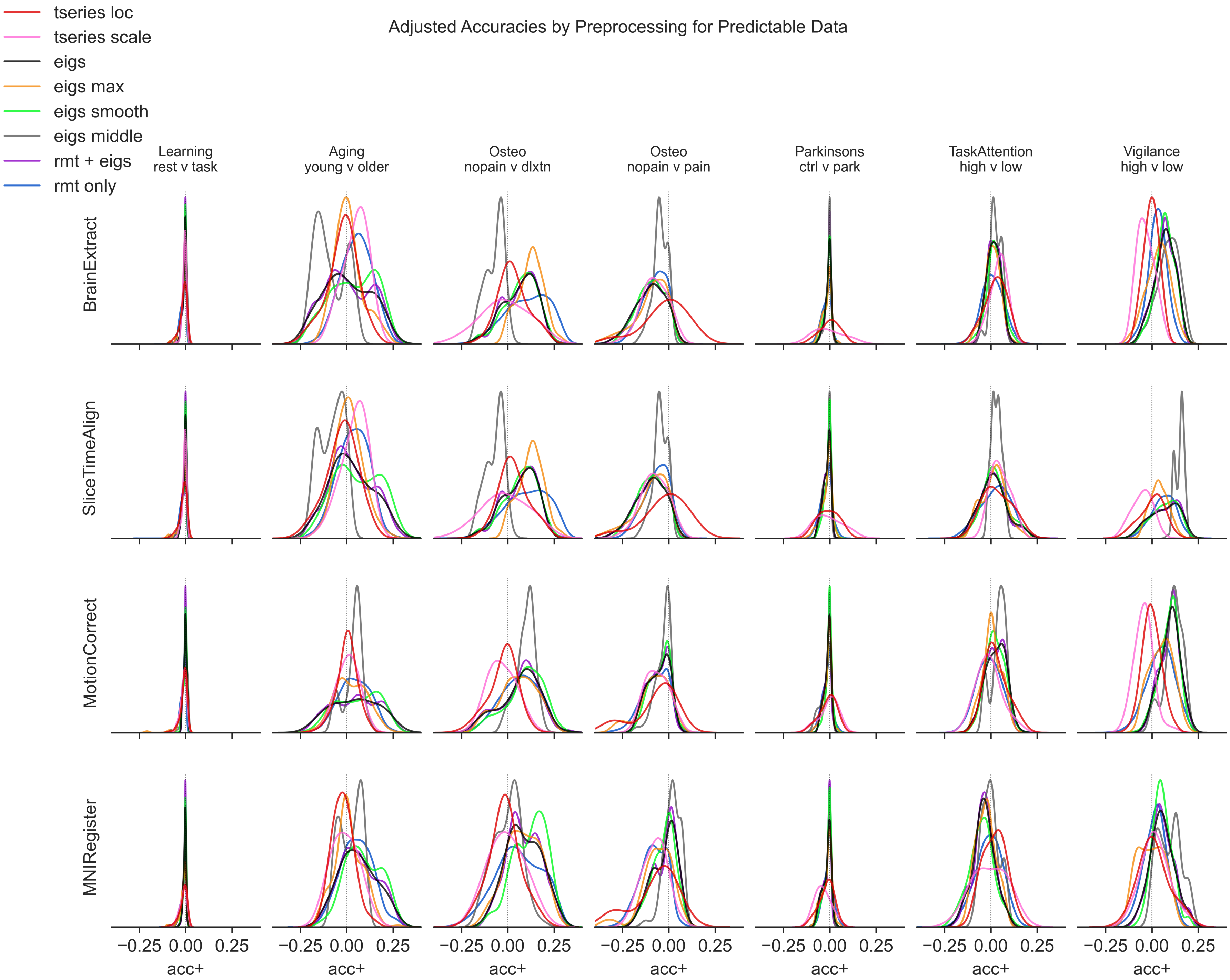

Figure 13: Adjusted accuracy distributions across fine feature groupings and predictable comparison tasks, with effect of preprocessing.

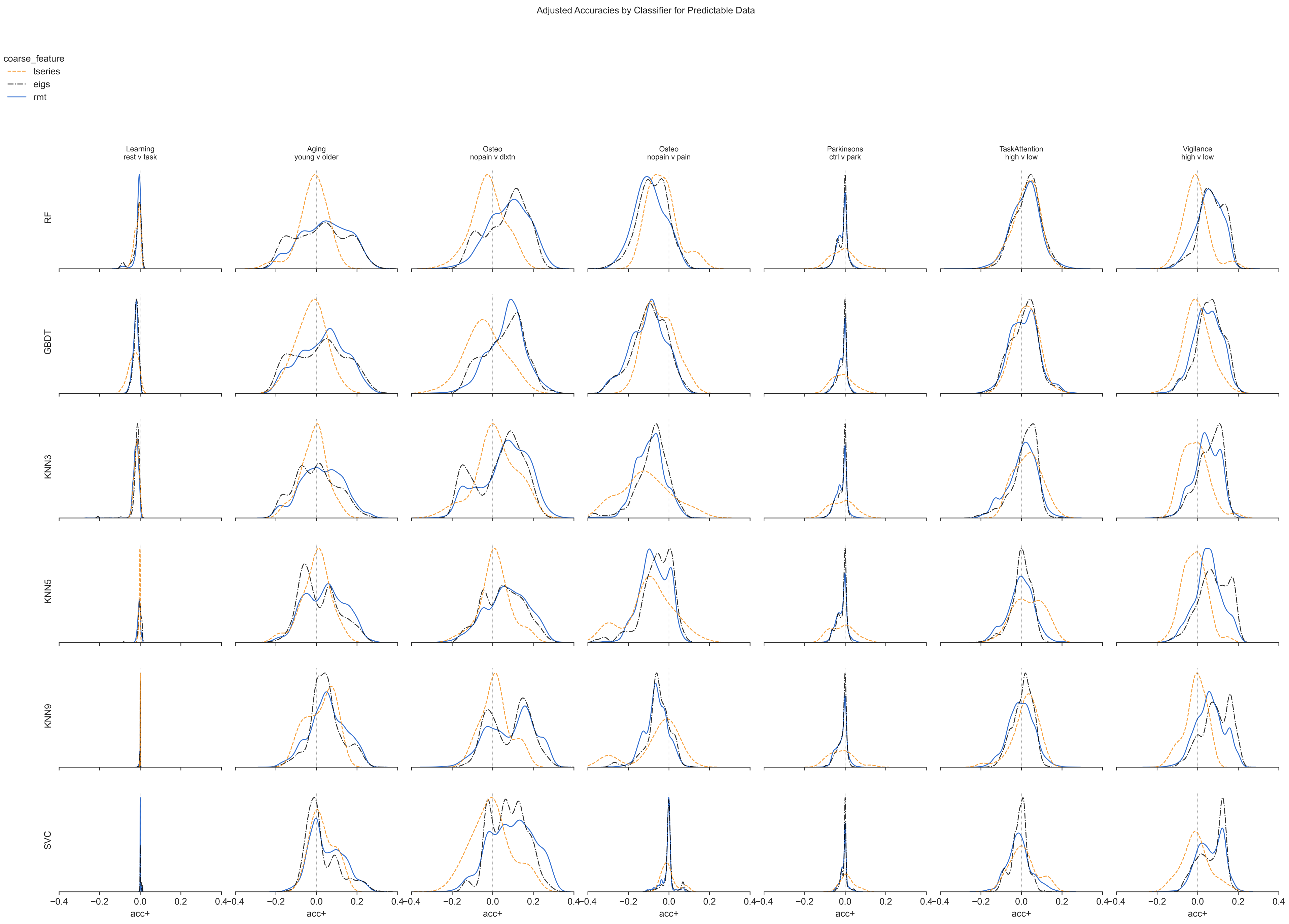

Figure 14: Adjusted accuracy distributions across coarse feature groupings and predictable comparison tasks, by classifier. Note the general similarity of each distribution within a particular classification task (column) and within each feature grouping.

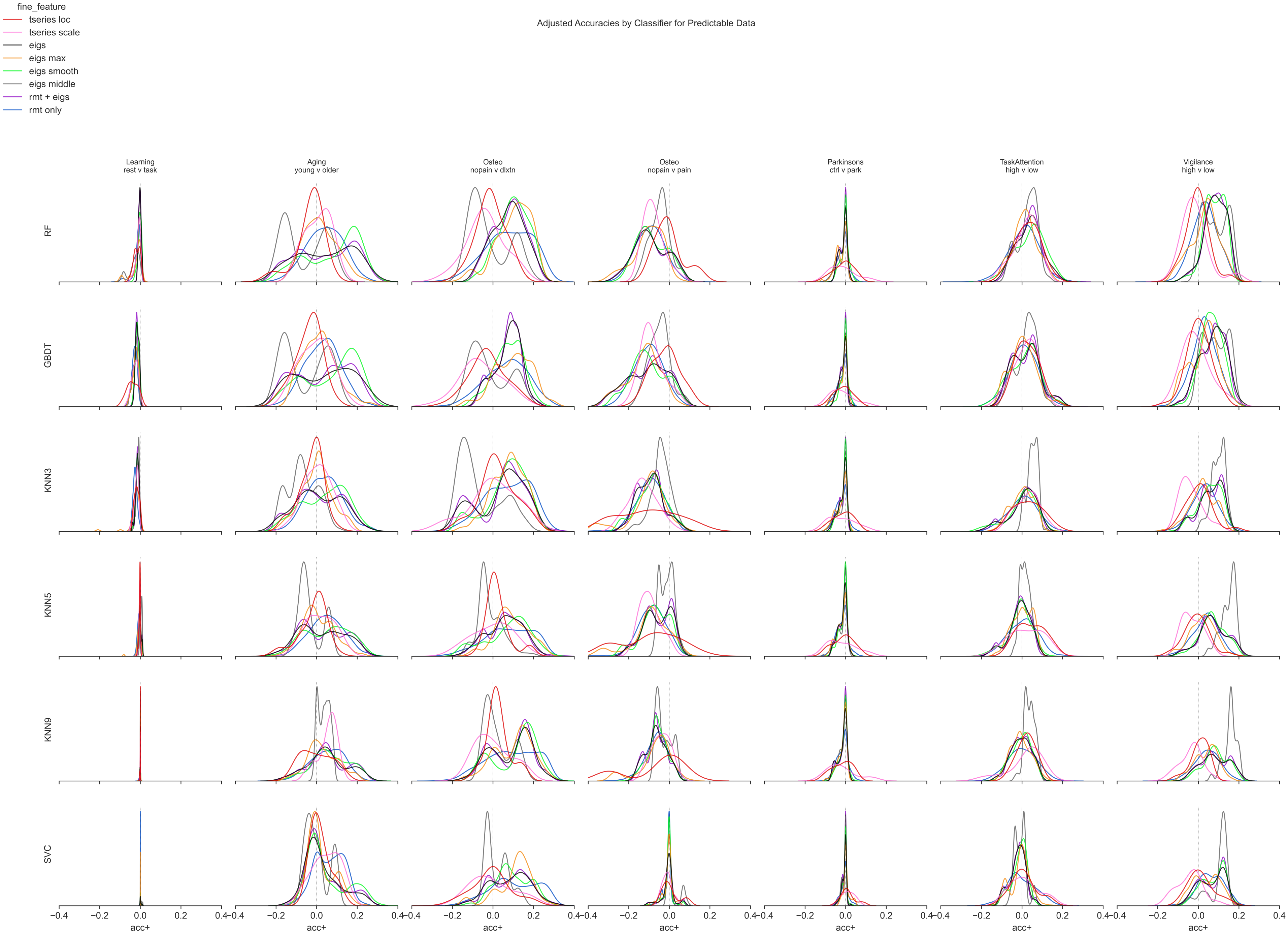

Figure 15: Adjusted accuracy distributions across fine feature groupings and predictable comparison tasks, by classifier. Note the general similarity of each distribution within a particular classification task (column) and within each feature grouping. Note also that, within a classification task (column), that the rank ordering of features, based on wither the median, mode, or mean, does not change dramatically or consistently from classifier to classifier.

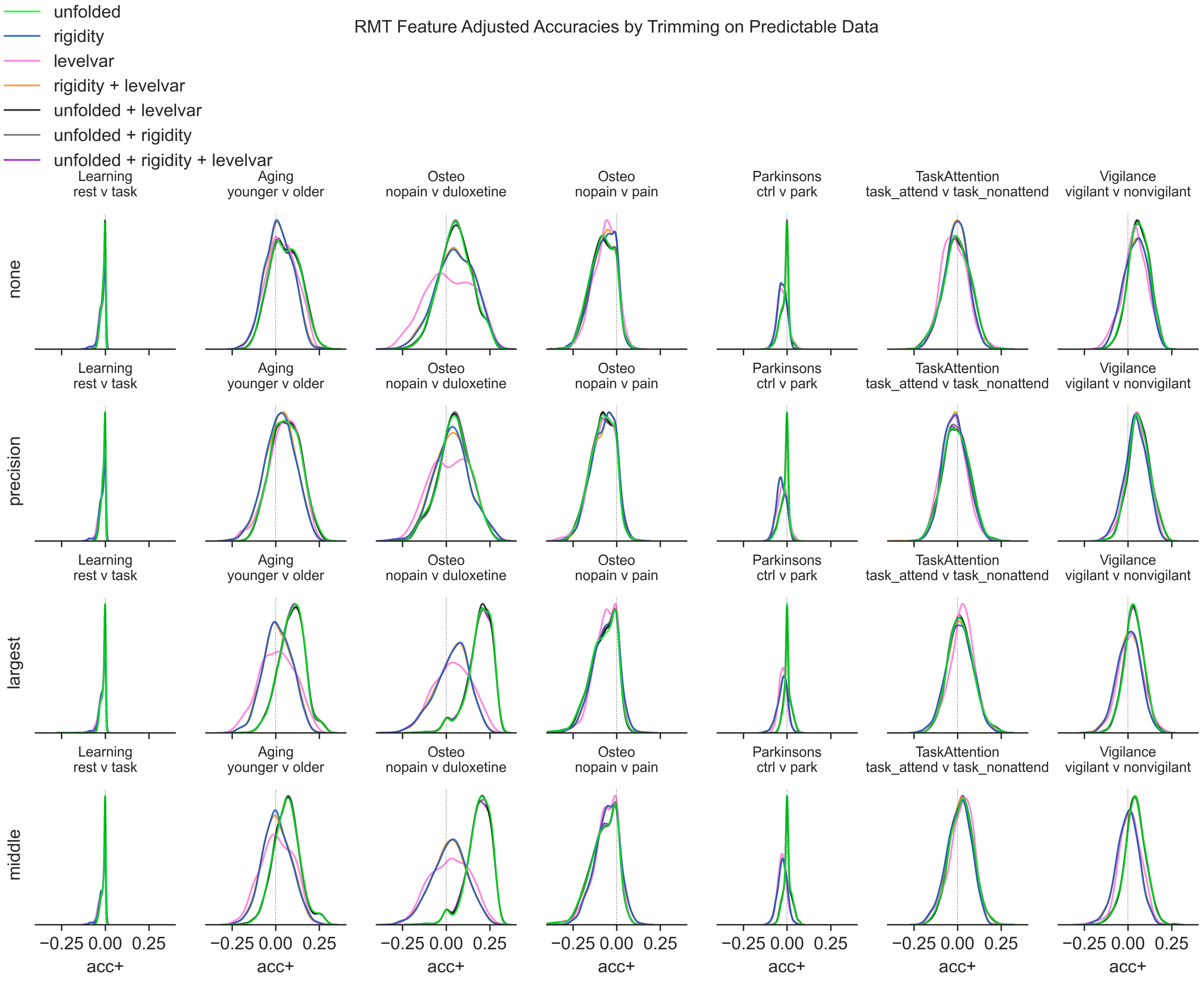

Figure 16: Distributions of mean adjusted accuracies for unfolding-dependent RMT features, by trimming. Note the tendency for a rightward shift in the distributions of the features involving the unfolded eigenvalues when using largest or middle trimming (most dramatic in the Osteo nopain v duloxetine condition). The impact of these trimming methods on the rigidity and level variance features, however, was mixed (compare Vigilance data to Osteo nopain v duloxetine condition.)

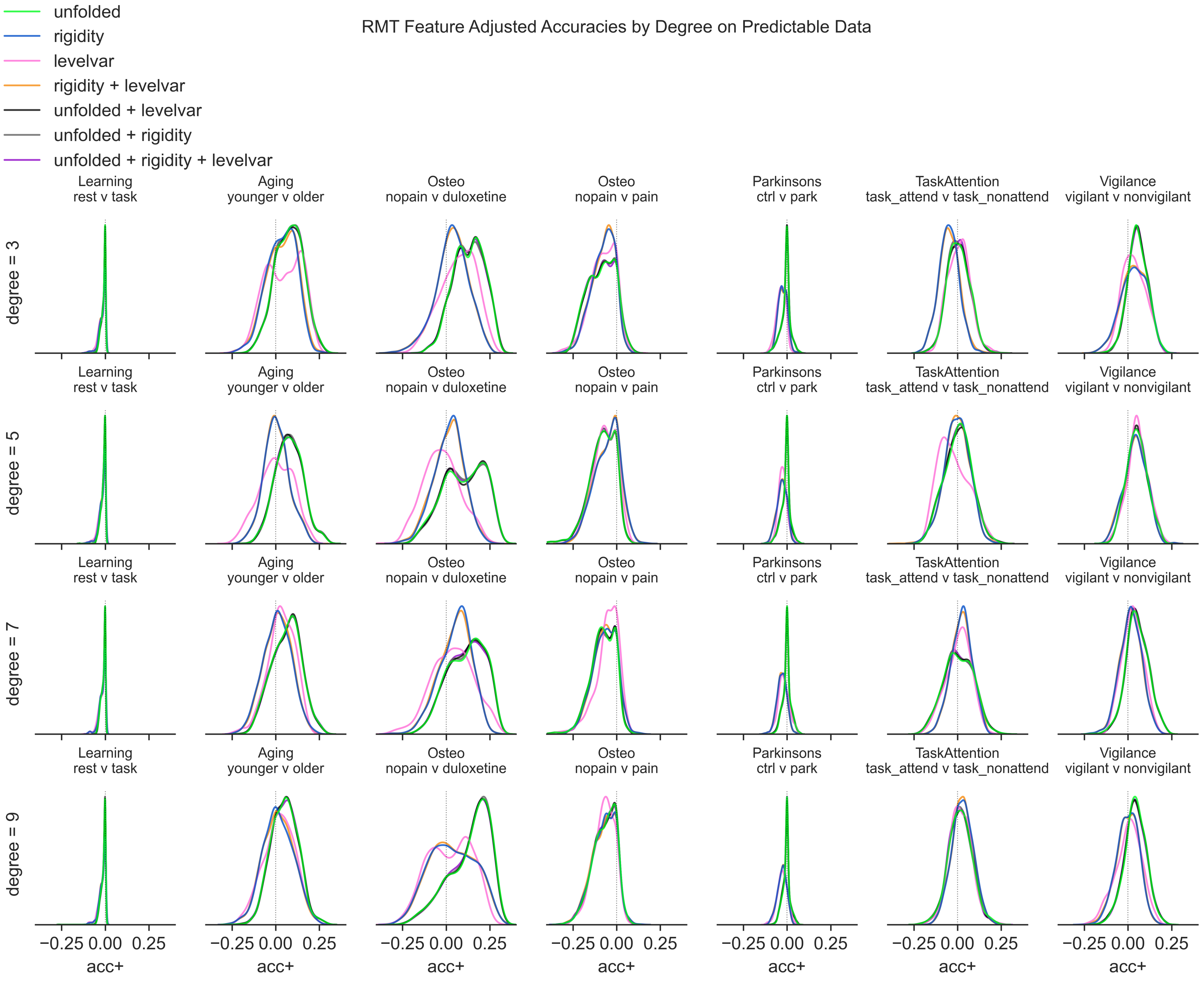

Figure 17: Distributions of mean adjusted accuracies for unfolding-dependent RMT features, by degree.

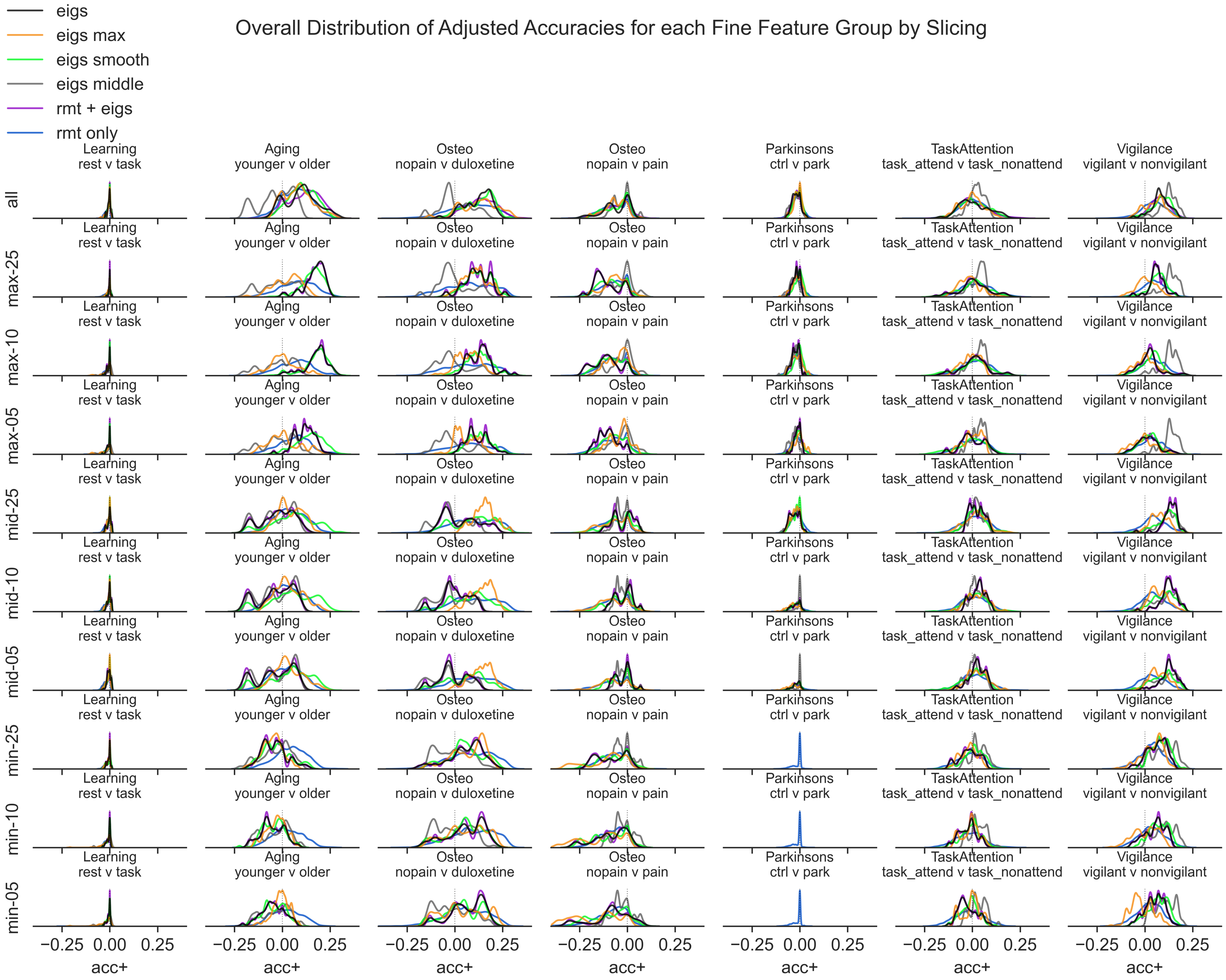

Figure 18: Distributions of mean adjusted accuracies by slicing. Features involving the full spectrum (raw eigenvalues, smoothed eigenvalues, and rmt + eigs) sometimes have most positive adjusted accuracy distributions when using the larger eigenfeature values (first two columns) or middle values (Osteo nopain v pain condition, Vigilance classification task).

Feature	mean	min	5%	50%	95%	max	std
unfolded	0.021	-0.437	-0.123	0.000	0.196	0.312	0.091
unfolded + levelvar	0.021	-0.437	-0.122	0.000	0.195	0.312	0.091
unfolded + rigidity	0.021	-0.437	-0.122	0.000	0.194	0.312	0.091
unfolded + rigidity + levelvar	0.021	-0.437	-0.123	0.000	0.193	0.312	0.090
eigs + eigs_smooth	0.025	-0.295	-0.125	0.000	0.193	0.337	0.091
eigs + savgol	0.022	-0.295	-0.125	0.000	0.190	0.337	0.091
eigs + unfolded	0.014	-0.311	-0.130	0.000	0.173	0.362	0.087
eigs + rigidity + levelvar	0.013	-0.311	-0.130	0.000	0.173	0.362	0.086
eigs + unfolded + levelvar	0.013	-0.311	-0.130	0.000	0.173	0.362	0.087
eigs + unfolded + rigidity	0.013	-0.311	-0.130	0.000	0.173	0.362	0.086
eigs_savgol	0.016	-0.313	-0.126	0.000	0.172	0.321	0.085
eigs_smooth	0.018	-0.263	-0.123	0.000	0.172	0.323	0.084
eigs + levelvar	0.013	-0.311	-0.131	0.000	0.171	0.321	0.086
eigs	0.013	-0.311	-0.130	0.000	0.168	0.312	0.086
eigs + rigidity	0.013	-0.311	-0.131	0.000	0.162	0.312	0.085
eigsminmax20	0.007	-0.348	-0.123	0.000	0.159	0.309	0.083
eigsminmax5	0.002	-0.367	-0.120	0.000	0.159	0.284	0.084
T-p05	-0.004	-0.298	-0.193	0.000	0.155	0.205	0.091
eigsmiddle40	0.008	-0.225	-0.114	0.000	0.151	0.234	0.074
eigsmiddle20	0.006	-0.210	-0.120	0.000	0.147	0.215	0.074
eigsminmax10	0.003	-0.367	-0.116	0.000	0.145	0.287	0.081
levelvar	-0.004	-0.363	-0.135	-0.010	0.145	0.312	0.081
T-rrng	0.002	-0.249	-0.140	0.000	0.144	0.184	0.079
eigsmiddle10	0.004	-0.224	-0.127	0.000	0.136	0.206	0.074
rigidity + levelvar	-0.006	-0.335	-0.127	-0.009	0.130	0.312	0.076
rigidity	-0.006	-0.332	-0.127	-0.009	0.130	0.309	0.075
T-mean	0.011	-0.202	-0.080	0.009	0.119	0.191	0.058
T-med	-0.006	-0.259	-0.102	-0.002	0.114	0.194	0.065
T-max	-0.003	-0.192	-0.106	-0.002	0.112	0.180	0.066
T-rng	-0.004	-0.173	-0.104	-0.002	0.110	0.169	0.063
T-iqr	-0.030	-0.313	-0.152	-0.025	0.103	0.202	0.077
T-std	-0.025	-0.202	-0.126	-0.026	0.094	0.147	0.065
T-p95	-0.011	-0.228	-0.115	-0.002	0.079	0.180	0.061
T-min	-0.030	-0.298	-0.298	-0.001	0.047	0.127	0.082

Table 1: Numerical summaries of feature mean accuracy difference from guess across predictable comparisons, and all combinations of analytic choices, sorted by 95% percentile (robust max) value.
